# The role of the *SLC6A3* 3’ UTR VNTR in nicotine effects on cognitive, affective, and motor function

**DOI:** 10.1007/s00213-021-06028-x

**Published:** 2021-12-02

**Authors:** Rebekka Schröder, Martin Reuter, Kaja Faßbender, Thomas Plieger, Jessie Poulsen, Simon S. Y. Lui, Raymond C. K. Chan, Ulrich Ettinger

**Affiliations:** 1 Department of Psychology, University of Bonn, Bonn, Germany; 2 Nicotine Science Center, Fertin Pharma A/S, Vejle, Denmark; 3 Department of Psychiatry, The University of Hong Kong, Hong Kong Special Administrative Region, China; 4 Neuropsychology and Applied Cognitive Neuroscience (NACN) Laboratory, CAS Key Laboratory of Mental Health, Institute of Psychology, Beijing, China; 5 Department of Psychology, University of Chinese Academy of Sciences, Beijing, China

**Keywords:** Nicotine, SLC6A3, DAT, Smooth pursuit, Inhibition, Spontaneous blink rate, Proactive inhibition, Stop signal task, Individual differences

## Abstract

**Rationale:**

Nicotine has been widely studied for its pro-dopaminergic effects. However, at the behavioural level, past investigations have yielded heterogeneous results concerning effects on cognitive, affective, and motor outcomes, possibly linked to individual differences at the level of genetics. A candidate polymorphism is the 40-base-pair variable number of tandem repeats polymorphism (rs28363170) in the *SLC6A3* gene coding for the dopamine transporter (DAT). The polymorphism has been associated with striatal DAT availability (9R-carriers > 10R-homozygotes), and 9R-carriers have been shown to react more strongly to dopamine agonistic pharmacological challenges than 10R-homozygotes.

**Objectives:**

In this preregistered study, we hypothesized that 9R-carriers would be more responsive to nicotine due to genotype-related differences in DAT availability and resulting dopamine activity.

**Methods:**

*N*=194 non-smokers were grouped according to their genotype (9R-carriers, 10R-homozygotes) and received either 2-mg nicotine or placebo gum in a between-subject design. Spontaneous blink rate (SBR) was obtained as an indirect measure of striatal dopamine activity and smooth pursuit, stop signal, simple choice and affective processing tasks were carried out in randomized order.

**Results:**

Reaction times were decreased under nicotine compared to placebo in the simple choice and stop signal tasks, but nicotine and genotype had no effects on any of the other task outcomes. Conditional process analyses testing the mediating effect of SBR on performance and how this is affected by genotype yielded no significant results.

**Conclusions:**

Overall, we could not confirm our main hypothesis. Individual differences in nicotine response could not be explained by rs28363170 genotype.

**Supplementary Information:**

The online version contains supplementary material available at 10.1007/s00213-021-06028-x.

## Introduction

Nicotine is a non-selective agonist of the nicotinic acetylcholine receptors (nAChr; de Kloet et al. 2015; Wonnacott et al. 2005) that stimulate dopamine release in the nucleus accumbens (and striatum in general) through activation of dopamine neurons in ventral tegmental area (VTA; Bonci et al. 2003; Cachope et al. 2012; de Kloet et al. 2015; Nisell et al. 1994; Threlfell et al. 2012; Wonnacott et al. 2005).

Nicotine has been widely studied for its potential pro-cognitive effects (Hahn 2015; Heishman et al. 2010), especially in groups with attentional dysfunction such as patients with neurodegenerative diseases, schizophrenia and ADHD (Barr et al. 2008b; Barreto et al. 2014; D’Souza and Markou, 2012; Levin et al. 1996; Rezvani and Levin 2001). However, nicotine effects on cognitive performance in healthy individuals are heterogeneous, with some studies providing evidence for beneficial effects, specifically in the domain of attention, yet others suggest detrimental effects (Almeida et al. 2020; Ettinger et al. 2017; Hahn 2015; Heishman et al. 2010; Niemegeers et al. 2014; Wignall and de Wit 2011).

Generally, nicotine effects appear to depend on factors such as baseline performance, dosage and smoking status (Almeida et al. 2020; Niemegeers et al. 2014; Wignall and de Wit 2011), suggesting substantial interindividual variance in dopamine-related function.

There is also evidence that such interindividual variability may be linked to differences at the level of genetics (Hariri 2009; Siebner et al. 2009). Here, to explain variability in nicotine response, we focus on a 40-base pair variable number of tandem repeats (VNTR) polymorphism (rs28363170) in the 3’ untranslated region of the gene (*SLC6A3*) coding for the dopamine transporter (DAT). The DAT plays a major role in dopamine neurotransmission by controlling re-uptake of dopamine into the presynaptic neuron, thereby regulating synaptic dopamine availability (Piccini 2003; Salatino-Oliveira et al. 2018). DAT density is particularly high in striatum, a structure known to play a crucial role in dopamine response to nicotine (Cachope et al. 2012; Piccini 2003; Threlfell et al. 2012).

In humans, the most common alleles of the VNTR are the 9 (9R) and 10 repeat (10R) forms (Kang et al. 1999). At the behavioural level, there is no evidence of rs28363170 as a significant predictor of cognitive function in healthy adults (Gurvich and Rossell 2014; Rincón-Pérez et al. 2018). For outcomes both at the level of brain function and subjective experience, however, differences between genotypes have been identified in response to dopamine agonistic interventions (Brewer et al. 2015; Franklin et al. 2009, 2011; Gelernter et al. 1994; Kambeitz et al. 2014; Lott et al. 2005; Millar et al. 2011). These studies suggest that 9R-carriers are more responsive to challenges or interventions known to increase extracellular dopamine availability than 10R-homozygotes. However, the mechanisms of this effect are unclear. Here, we argue that differences in response between 9R carriers and 10R-homozygotes stem from differences in baseline DAT availability. Specifically, there is evidence for 9R carriers to have higher DAT availability (for meta-analysis, see Faraone et al. 2014), although this relationship was not significant in all studies (Kasparbauer et al. 2015; Wagner et al. 2014). This may be expected to result in lower baseline levels of extracellular dopamine in striatum.

In this preregistered study, we challenged the dopamine system by administering nicotine to rs28363170 9R carriers and 10R homozyogtes. We recorded spontaneous blink rate (SBR) as an indirect measure of striatal dopamine activity (Depue et al. 1994; Jongkees and Colzato 2016) to better understand possible genotype-related between-group differences in dopamine activity.

In order to characterise the interactive effects of nicotine and *SLC6A3-*genotype on cognitive, motor and affective functioning, we selected four paradigms that have been shown to be sensitive to dopaminergic influences. Specifically, we assessed smooth pursuit eye movements (SPEM; Meyhöfer et al. 2019), reactive inhibition in the stop signal task (Logemann et al. 2014a; Logan and Cowan 1984), proactive inhibition by comparing reaction times to go stimuli in the stop signal task and in a simple choice task where no stop signals are presented and effortful behaviour associated with affective processing in the Anticipatory and Consummatory Pleasure task (ACP; Heerey and Gold 2007; Lui et al. 2016).

In line with previous research, we expected 9R carrier to respond more strongly to nicotine administration than 10R homozygotes. We further hypothesized that drug effects on task performance are mediated by effects on SBR and that this relationship is moderated by genotype.

## Materials and methods

The study was approved by the ethics committee of the Faculty of Medicine at the University of Bonn (registration number 215/18). The study was preregistered at https://osf.io/6wux4. This preregistration entails that our research questions and analysis plans were defined and time-stamped prior to data collection (Nosek et al. 2018).

### Participants and screening procedure

We aimed for 200 participants to complete the study. This sample size yields at least 90% power to detect an effect of *f* = 0.25 with an alpha-level of .05 (G*Power 3.1.9.2; Faul et al. 2007).

We included healthy female and male non-smokers (at most 10 cigarettes in a lifetime, or equivalents such as e-vaping, nicotine gums etc.), aged 18-40 years, with normal or corrected to normal vision and carriers of 9R/9R, 9R/10R or 10R/10R genotypes. For statistical analyses, 9R-carriers (9R/9R, 9R/10R) were compared to 10R-homozygotes. A full list of exclusion criteria is available in Table 1.

**Table 1:** Inclusion and exclusion criteria

Inclusion criteria	Exclusion criteria
-Healthy -Male or female -18–40 years of age -Non-smoker (less than 10 cigarettes in lifetime) -Carrier of the 9R/9R, 9R/10R or 10R/10R genotype -Normal or corrected-to-normal vision -Good German skills	-Known allergic reaction to nicotine -Known heart disease -Known brain circulatory disorder (e.g., stroke) -Hypertension (systolic ≥140 and diastolic ≥90) -Hypotension (systolic <100 and diastolic <60) -Bradycardia (resting pulse <60 per minute) -Tachycardia (resting pulse >100 per minute) -Known circulatory disorder -Known diabetes mellitus -Known hyperthyreosis -Known tumour in the adrenal gland -Known kidney or liver disease -Known oesophagitis, infections in mouth or throat, gastritis or stomach ulcers -Known fructose intolerance -Body mass index (BMI) <18 or >29 for men or <19 or >30 for women -For women: not using effective contraceptives for at least one cycle, pregnant or breastfeeding -Current drug abuse -Current medical or CNS disease -Current psychiatric or neurological diagnoses -Current medication intake (except oral contraceptives or vitamin preparations) -Current participation in a medication trial

Participants were recruited via advertisements on the campus of the University of Bonn, circular emails and social media. They were invited to fill in a short online questionnaire to confirm basic inclusion criteria. Suitable participants were invited to an in-person screening at the Department of Psychology at the University of Bonn. In the screening, participants confirmed their willingness to participate in the study and provided written, informed consent. Then, a semi-structured interview was conducted to screen for psychiatric (Ackenheil et al. 1999), neurological or physical disorders and further exclusion criteria (Table 1). Handedness (Oldfield 1971), verbal intelligence (Lehrl 1999), blood pressure, heart rate, height and weight were obtained and body mass index (BMI) was calculated. Finally, participants provided a DNA sample (see below).

Suitable participants were then invited to the experimental assessment. They were asked to arrive well rested at the laboratory and to abstain from alcohol and medication at least 24 hours before the assessment and from citrus fruits on the day of the assessment. In addition, they were asked to maintain their usual caffeine intake and to have a light meal before the assessment.

### Study design and procedure

The study followed a double-blind, placebo-controlled randomized between-subjects design, with separate randomization for females and males. The study team carrying out the assessments were involved neither in generating the randomisation list nor in the preparation of the nicotine and placebo gums.

At the beginning of each assessment, inclusion and exclusion criteria were reconfirmed. Then, participants were asked to provide a urine sample for analyses of current use of nicotine (qualitative cotinine tests with a 200 ng/ml cut-off, nal von minden GmbH, Moers, Germany) and pregnancy (Runbio Biotech Co., Guangdong, China; female participants only). Positive results led to study exclusion.

Participants were then given a chewing gum containing either 2 mg nicotine (Nicotinell®, spearmint) or placebo (Fertin Pharma, Vejle, Denmark). The placebo gums were customized to match the taste, mouth feel and appearance of the nicotine gums as accurately as possible. The gum was chewed following a standardized protocol (Meyhöfer et al. 2019). Voice-recorded instructions presented via headphones asked participants to alternate between chewing and keeping the gum between upper front teeth and lips for 30 minutes (12 short periods of each). Immediately after completion of the chewing protocol, participants filled in computerised visual analogue rating scales (VAS; Bond and Lader 1974) to assess subjective feelings. Blood pressure and heart rate were measured.

Then, SBR was assessed. Subsequently, the SPEM, ACP, stop signal and simple choice tasks were carried out in randomized order. However, the stop signal and simple choice tasks were always presented as a block, starting with the stop signal task for half of the participants and the simple choice task for the other half. At the end of each assessment, participants were asked to guess whether they had received nicotine or placebo.

**Fig. 1:**
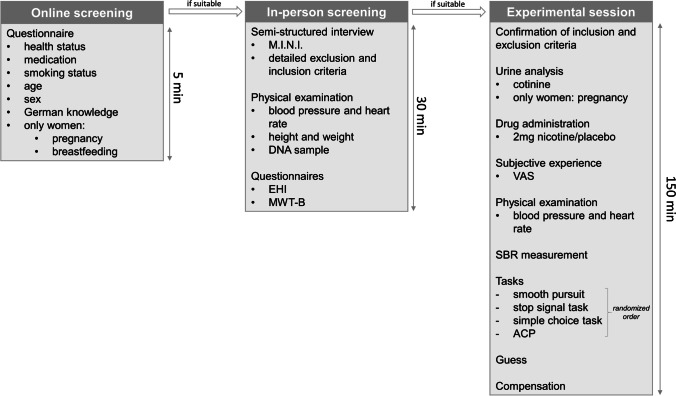
Study procedure. M.I.N.I: Mini International Neuropsychiatric Interview (Ackenheil et al., 1999), EHI: Edinburgh Handedness Inventory (Oldfield, 1971), MWT-B: Mehrfachwahl-Wortschatz-Intelligenztest, version B (Lehrl, 1999), VAS: visual analogue scales (Bond & Lader, 1974), SBR: spontaneous blink rate, ACP: Anticipatory and Consummatory Pleasure Task.

Participants received course credits or €30 for participating. The experimental session took approximately 2 hours 30 minutes. An overview of the study procedure is depicted in Figure 1.

### DNA extraction and genotyping

All participants provided buccal mucosa cell samples for DAT genotyping. DNA was extracted using commercial MagNA Pure extraction kits (MagNA Pure LC DNA isolation kit; Roche Diagnostics; Mannheim, Germany).

Amplification of the DAT VNTR was conducted by means of polymerase chain reaction (PCR). Primer sequences for amplification were 5’-TGTGGTGTAGGGAACGGCCTGAG-3’ and

5’-CTTCCTGGAGGTCACGGCTCAAGG-3’. The PCR protocol started with 3 minutes of initial denaturation at 94°C followed by 39 cycles of 45 seconds denaturation at 94°C, 30 seconds annealing at 62°C, and 30 seconds extension at 72°C. The final elongation at 72°C lasted 5 minutes. PCR products were genotyped by electrophoresis on a 2% agarose gel in a TBE solution and subsequent visualization under UV light.

Genotype frequencies of the DAT VNTR (9R/9R: *N* =14; 9R/10R: *N* = 80; 10R /10R: *N* = 100) were in Hardy Weinberg equilibrium (χ^2^ = 0.14, *p* = .713).

### Tasks

The fixation and SPEM tasks were built in ExperimentBuilder (SR Research Ltd., Ontario, Canada, version 1.10) and presented on a 24-inch BenQ LCD monitor (resolution 1920×1080 px; 120 Hz refresh rate). To record eye movements and blink rate, a desktop-mounted video-based combined pupil and corneal reflection eye-tracker (EyeLink 1000, SR Research Ltd.) was used. A centroid pupil-tracking algorithm was employed to detect pupil and corneal reflection of the right eye at 1000 Hz sampling rate. Prior to each task, a five-point horizontal-vertical calibration was performed. During the tasks, participants rested their head on a chin-rest.

The ACP, stop signal and simple choice tasks were presented on a 19-inch Hyundai LCD monitor (resolution 1440×900 px, 60 Hz refresh rate).

Distance from eye to monitor was approximately 70 cm for all tasks and instructions and stimuli were presented on a black (0, 0, 0) screen.

#### Fixation

The fixation target was a grey (128, 128, 128) circle (diameter = 15px/0.27°, stroke width = 5px/0.09°) presented at the centre (0°, 0°) of the screen for 180 seconds. Participants were instructed to fixate on the target as accurately as possible with their eyes while keeping their head still.

SBR (N/s) was obtained using DataViewer (SR Research Ltd.).

#### Smooth pursuit

The smooth pursuit task (Supplementary Figure 1) was the same as the one used by Meyhöfer et al. (2019). The task was presented on the inner 1680×1050 px of the monitor. Surrounding pixels were black. The smooth pursuit target was a grey (128, 128, 128) circle (diameter = 15px/0.27°, stroke width = 5px/0.09°) moving horizontally between ±432px (7.89°) across the screen in a sinusoidal velocity waveform at three target velocities (or frequencies: 0.2 Hz, 0.4 Hz, 0.6 Hz). The sinusoidal pattern indicates that target velocity constantly changed over time, accelerating towards the center of the screen, and decelerating towards the turning points. Peak and average velocities were 9.91°/s and 6.31°/s for the 0.2 Hz target, 19.83°/s and 12.62°/s for the 0.4 Hz target and 29.74°/s and 18.94°/s for the 0.6 Hz target. Each target velocity was presented twice, once with and once without a stationary structured background, resulting in a total of 6 blocks. The structured background consisted of a symmetrical six-by-six grid of white (255, 255, 255) circles (diameter = 15px/0.27°, stroke width = 5px/0.09°) with the following corner coordinates in pixels: 408, 310; 408, 740; 1272, 310; 1272, 740 (in inner 1680×1050 px of the monitor). Each block was presented for 30 seconds in randomized order. Participants were instructed to follow the target as accurately as possible with their eyes while keeping their head still. A brief practice task was presented prior to the task, consisting of four blocks (0.4 Hz without background, 0.2 Hz with background, 0.4 Hz with background, 0.6 Hz without background), each lasting five seconds.

Eye movement data were preprocessed in Matlab R2016A (Natick, Massachusetts: The MathWorks Inc.). First, the first excursion of the target from the centre, blinks and saccades were excluded. Then, segments of pursuit in the middle 50% of each half-cycle lasting 50 ms or longer were identified. Velocity gain was the primary outcome measure of pursuit performance, calculated as the time-weighted average of the ratio of mean eye velocity to mean target velocity for these segments. Optimal performance corresponds to a gain value of one.

For both eye-tracking tasks, data quality was first individually assessed. Participants with poor eye-tracking data quality were excluded from analyses.

#### Stop signal task

The stop signal task (Supplementary Figure 2) was written using Presentation® software (Version 18.0, Neurobehavioral Systems, Inc., Berkeley, CA). It was adapted from the stop signal task provided in the Cognitive Experiment III v3 pack provided by Neurobehavioral Systems (www.neurobs.com). The task consisted of 150 go trials and 50 stop trials. In go trials, participants had to indicate the direction of a centrally presented arrow (go stimulus, “<” or “>”) by pressing a key on a ‘qwertz’ keyboard (“x” and “;”, respectively). In stop trials, a stop stimulus (“⋀”) was presented immediately after the go stimulus. In these trials, participants had to inhibit their responses.

Each trial started with a fixation cross presented centrally for 500 ms. Then, the go-stimulus appeared at the same position for 100 ms followed by a blank screen. In stop trials, the stop stimulus appeared after the current stop signal delay (SSD) for 500 ms. In go trials, a black screen was presented until the next trial was initiated (current SSD-100ms+500ms). The intertrial interval was 1000 ms (black screen). The initial SSD of 400 ms was adjusted to the participant’s performance (maximum 500 ms, minimum 50 ms) in 16 ms steps using a tracking procedure (Verbruggen et al. 2019). Thus, SSD was increased by 16 ms after successful stop trials and decreased by 16 ms after unsuccessful stop trials, converging on a 50% probability of successful stop trials. If the SSD was shorter than 100 ms, the go stimulus was presented for the duration of the SSD. Stop trials occurred equally often after right and left arrows, respectively. Trial order was randomized.

All cues were presented in Helvetica font in white (255, 255, 255) on black (0, 0, 0) background. Font sizes were 7.5% of screen height for the fixation cross, 10% for go stimuli and 12.5% for stop stimuli.

Participants were instructed to leave their index fingers on the response keys throughout the entire task. They were asked to respond as fast and accurately as possible and not to wait for the stop signal.

Prior to the task a practice block (20 trials) was presented. If the accuracy in this block was less than 50%, it was repeated until an accuracy of more than 50% was achieved.

Stop signal reaction time (SSRT) was obtained as the primary outcome measure. It was calculated using the integration method with replacement of go-omissions (Verbruggen et al. 2019).

Participants were excluded if go trial accuracy was less than 80%, and/or if they had more than 75% or less than 25% successful stop-trials and/or in case of negative SSRT (Congdon et al. 2012; Verbruggen et al. 2019).

#### Simple choice task

The simple choice task (Supplementary Figure 3) was identical to the stop signal task with the difference that no stop trials were presented. Hence, participants had to respond to left and right arrows with the “x” and “;” keys, respectively, in 150 trials (75 right, 75 left). After each go stimulus a black screen was presented for 900 ms followed by the intertrial interval of 1000 ms. Instructions were to respond as fast and accurately as possible and to leave the index fingers on the response keys throughout. Again, 20 practice trials were presented before the task and repeated until accuracy exceeded 50%.

To assess proactive inhibition, reaction times of correct go responses were obtained both for the stop signal task and the simple choice task.

Participants were excluded if their go-accuracy in either task was less than 80%.

#### ACP

The ACP task (Supplementary Figure 4) was written using E-Prime Software (Version 2.0, Psychology Software Tools, Sharpsburg, PA, USA). It was adapted from Lui et al. (2016) and comprised an anticipatory phase followed by a consummatory phase.

In the anticipatory phase, participants saw 42 slides showing three pictures each. The pictures were drawn from the International Affective Pictures System (Lang et al. 1997). Three types of slides were used (14 positive, 14 negative and 14 neutral). All pictures on one slide were from the same category. The slides were presented consecutively. First, participants were asked to rate valence and arousal evoked by each slide on a nine-point Likert scale, ranging from *very unpleasant* to *very pleasant* and *very calm* to *very arousing*, respectively. Ratings were obtained by the number keys at the top of the keyboard. Rating time was not limited, but slides were removed from the screen after rating. Second, participants were asked to indicate whether they wanted to see a particular slide again later or not. They were told they could alter the probability of the later reappearance of a slide by rapidly and alternately pressing two keys on the keyboard. Half of the participants were instructed to press “m” and “n” on the keyboard to increase the probability of seeing the current slide again and “x” and “y” to decrease the probability. For the other half of the participants, the key assignment was reversed. The response window for the key presses was two seconds. During this time, participants saw an instruction to press the keys and a reminder of the key allocation. Each trial started with a 500 ms fixation dot and ended on a 2000 ms rest period. Participants were instructed to use the index and middle fingers of their left and right hands. In addition, they were asked to only press the keys if they in fact wanted (or not wanted) to see a particular slide again and to not press the keys if they were indifferent to whether they wanted to see the slide again or not.

In the consummatory phase, participants saw 30 slides (10 positive, 10 negative, 10 neutral) from the anticipatory phase. They could alter the presentation time of the slides by rapidly and alternately pressing the same keys as in the anticipatory phase. Presentation times were prolonged if they pressed the keys used in phase 1 to increase slide probability (e.g., “m” and “n”). Presentation times were shortened if they pressed the keys used in phase 1 to decrease slide probability (e.g., “x” and “y”). Presentation times ranged from two to ten seconds depending on participants’ key press response. If no keys were pressed, presentation time was five seconds. Participants had no influence on total task duration as intertrial intervals (black screen) were adjusted to slide presentation durations so that total trial duration and intertrial interval were always the same.

A practice block was carried out prior to each phase.

The primary outcome measure was key pressing speed, i.e., the number of key presses per second (N/s), to account for differences in target presentation duration in the second task phase. Importantly, the factor valence was determined individually according to each participant’s valence ratings. Ratings of 1-3 were considered negative, 4-6 neutral and 7-9 positive.

A trial was considered invalid if a participant’s Likert rating of a slide did not match their key press response, e.g., if a participant gave a positive rating but pressed keys (> 4 key presses) to decrease probability of later stimulus reappearance or to shorten presentation duration, and vice versa for negative ratings. However, slides with neutral ratings were always valid. Trials with invalid responses were excluded from analysis. To account for individual differences in key pressing speed, results from a calibration block at the beginning of the task were applied in the consummatory phase in order to adjust presentation durations similarly between participants.

### Visual analogue scales

Subjective feelings after drug administration were assessed with computerised visual analogue scales (VAS; Bond and Lader 1974), yielding alertness (9 scales), calmness (2 scales) and contentedness (5 scales) factors. VAS scales were 100 mm long and ratings are reported as average values for each factor with higher values indicating higher expressions on the factors.

### Statistical analyses

For the primary outcome of each task, analysis of variance (ANOVA) and conditional process analysis were carried out. For subjective and cardiovascular outcomes, *t*-tests were carried out. Significance threshold for all analyses was α = .05. Departing from our preregistration, outliers were not winsorized following recent recommendations (Leys et al. 2019).

#### Analyses of variance

For each dependent variable (SBR, SPEM velocity gain, SSRT, go RT, key press speed), a between-subjects ANOVA with the factors drug (nicotine, placebo) and genotype (9R, 10/10) was carried out. Some analyses had additional within-subject factors, depending on the task analysed. For SPEM, the additional within-subjects factors were target velocity (0.2 Hz, 0.4 Hz and 0.6 Hz) and background (present, absent). For ACP, the additional within-subjects factors were valence (determined individually according to ratings; positive, negative and neutral) and phase (anticipatory, consummatory). The proactive inhibition analysis had the additional within-subjects factor task (stop signal, simple choice task).

Effect sizes were calculated as partial eta squared. If the sphericity assumption was violated, Greenhouse-Geisser correction was applied. Uncorrected degrees of freedom and Greenhouse-Geisser ε were obtained. Bonferroni-corrected *t*-tests were calculated as post hoc tests with *d*_av_ (Lakens 2013) as effect size for repeated-measures factors. Uncorrected *p*-values were obtained but significance was inferred from corrected alpha-thresholds.

#### Conditional process analyses

Based on our preregistered hypotheses, a conditional process analysis was performed for the primary outcome measures of each task (averaged across within-subject conditions for key press speed and SPEM velocity gain; difference between go and stop signal task for go reaction times) with the R *process* package (Hayes 2015). Specifically, model 8 was tested with four different outcome variables (Y; SPEM velocity gain, SSRT, go RT, key press speed). Drug was the independent variable (X), SBR was the mediator (M) and genotype was the moderator (W) on the paths between drug and SBR and drug and outcome measures, respectively. Bootstrap 95%-confidence intervals were calculated with 5000 bootstrap iterations. Participants were excluded according to above criteria. Specifically, a participant was not included in the conditional process analysis if fixation data quality was poor. Unstandardized regression coefficients are reported. Drug and genotype were coded as uncentered dichotomous variables (placebo = 0, nicotine = 1; 10/10 = 0, 9R = 1).

**Fig. 2:**
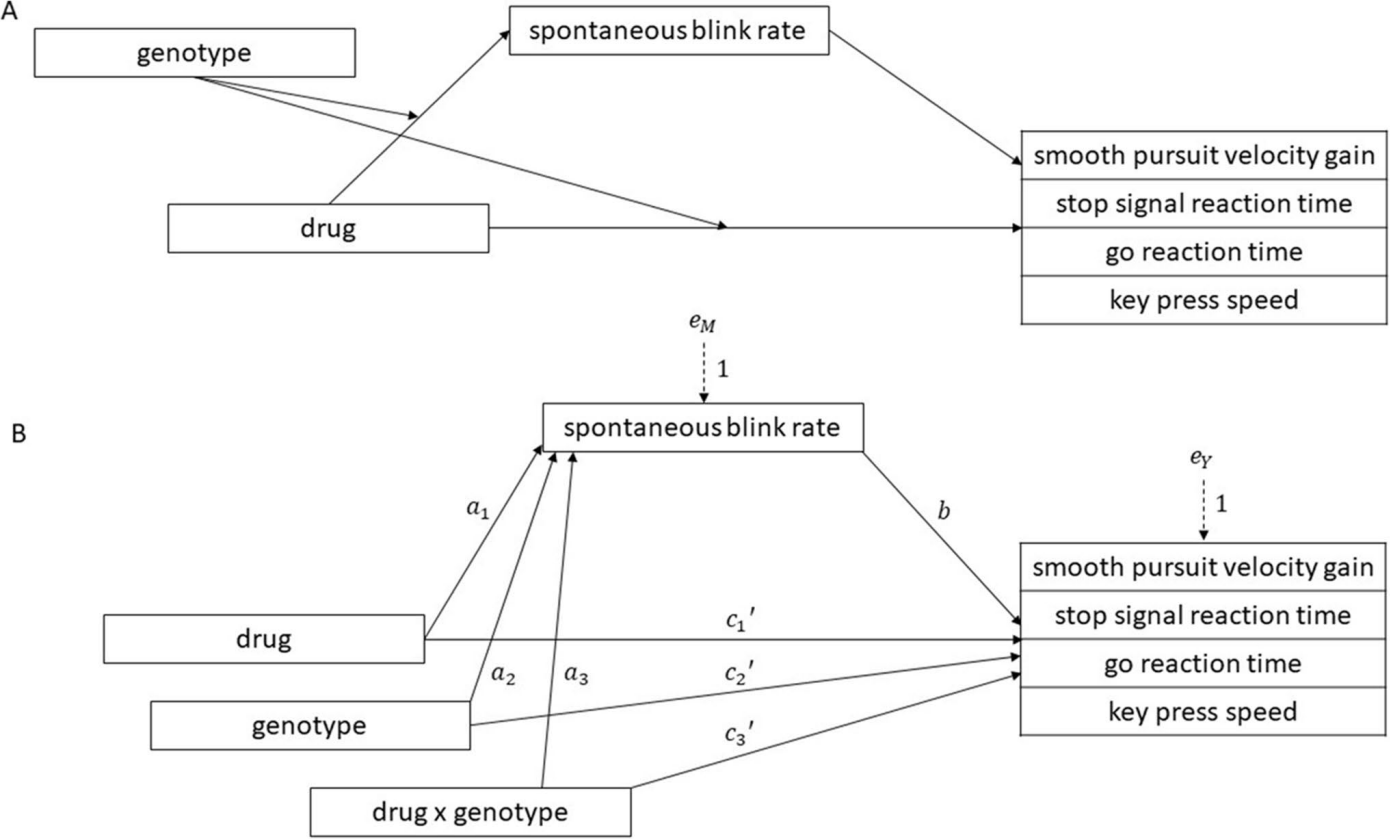
Conceptual (panel A) and statistical (panel B) diagram of the conditional process models. Spontaneous blink rate is considered a mediator of the drug effects on the four performance outcomes (smooth pursuit velocity gain, stop signal reaction time, go reaction time and key press speed). For each performance outcome, a separate analysis was carried out. Genotype acts as a moderator of the drug effect on spontaneous blink rate and performance.

Conceptual and statistical diagrams of the conditional process model are depicted in Figure 2.

#### Additional analyses

Two-sample *t*-tests were carried out in order to test drug effects on heart rate, blood pressure and the three VAS scales. Cohen’s *d* was calculated to determine effect sizes. A *χ*^2^-test of independence was calculated to assess whether participants guessed correctly if they had received nicotine or placebo.

#### Bayesian analyses

In addition to the preregistered analyses described above, Bayesian ANOVAs were calculated using JASP software (Version 0.14.1, JASP Team [2020]). Dependent and independent variables were selected as described in "Analyses of variance". JASP default priors were used, i.e. Cauchy priors centered on zero with a fixed effects scale factor of *r* = 0.5 and random effects scale factor of *r* = 1.

Bayes factors (BF) are interpreted according to Wagenmakers et al. (2018) with BF > 100 suggesting extreme evidence, 30-100 very strong evidence, 10-30 strong evidence, 3-10 moderate evidence, 1-3 anecdotal evidence and 1 no evidence. Bayesian model averaging (across all models) yielded BF quantifying the evidence for including or excluding a specific main or interaction effect (van den Bergh et al. 2020).

### Data and code availability

Anonymized data and analysis code are available at https://osf.io/bg3c6/.

## Results

### Participants

A total of 739 participants filled in the online questionnaire, 507 of whom met initial criteria and were invited to a face-to-face screening. Of those, 271 followed the invitation and 227 of them were considered suitable for participation. After genotyping, 18 participants had to be excluded because DNA analyses were inconclusive or revealed rare genotypes (not 9R/9R, 9R/10R or 10R/10R). Of the remaining participants, seven did not follow the invitation, four had positive cotinine tests, two consumed alcohol or took medication prior to testing and two had to discontinue participation due to adverse nicotine side effects.

The final sample consisted of *N* = 194 participants (153 females, 41 males), aged *M* = 22.82 years (*SD* = 3.33 years). Ninety-nine participants received nicotine (44 9R-carriers and 55 10R-homozygotes) and ninety-five participants received placebo (50 9R-carriers and 45 10R-homozygotes). Further demographic information is in Table 2.

**Table 2:** Demographic information and blink rates of the experimental groups

	Nicotine		Placebo	
	9R	10/10	9R	10/10
*N*	44	55	50	45
Age				
(mean, SD)	22.05 (2.53)	22.89 (3.30)	23.1 (3.76)	23.18 (3.52)
Gender (*N* females/ males)	37/7	40/15	40/10	36/9
Handedness (*N* right/left/ambidextrous)	35/7/2	47/6/2	44/6/0	35/7/3
Years spent in formal education (mean, SD)	15.39 (2.16)	15.98 (2.45)	15.82 (3.42)	15.98 (3.49)
MWT-B sum score (mean, SD)	25.18 (4.13)	26.18 (4.33)	25.24 (3.86)	25.13 (4.2)
SBR (N/s; mean, SD)	0.22 (0.43)	0.18 (0.18)	0.16 (0.14)	0.17 (0.19)

Legend: Demographic information and blink rates of the four experimental groups. MWT-B: Mehrfachwahl-Wortschatz-Intelligenz Test (Version B). Handedness was assessed with the Edinburgh Handedness Inventory. SBR: spontaneous blink rate.

Data collection took place between December 2018 and February 2020. It was discontinued before the targeted sample size of 200 was reached due to a nationwide lockdown related to the Sars-CoV-2 pandemic. However, we believe that the deviation is so minor that it does not have a significant impact on the statistical power in this study.

Due to poor eye-tracking data quality, two participants were excluded from SBR analyses and seven participants from SPEM analyses. Thirty-three participants were excluded from stop signal task and twelve participants from simple choice task analyses for fulfilling above exclusion criteria. Three participants were excluded from ACP analyses due to technical errors or failure to understand instructions.

### Fixation

**Fig. 3:**
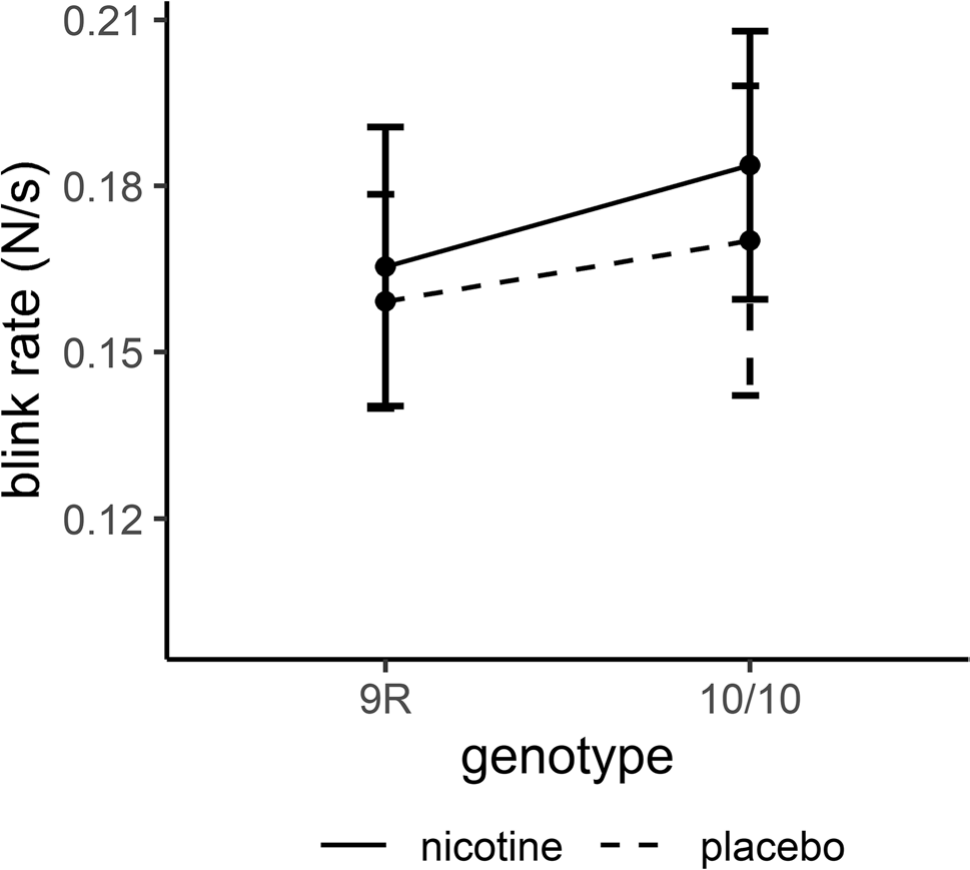
Effects of drug and genotype on spontaneous blink rate. Data are presented as mean ± standard errors. *N* = 192.

ANOVA on SBR yielded no significant main effects for drug or genotype and no interaction of the two factors (all *p* > .05; Table 2; Figure 3).

The Bayesian ANOVA suggested that the data were best represented by a null model (see Supplementary Table 1). There was moderate evidence to exclude drug (BF_excl_ = 8.34) and genotype (BF_excl_ = 7.62) and very strong evidence to exclude their interaction (BF_excl_ = 52.98) (see Supplementary Table 2).

### SPEM

**Fig. 4:**
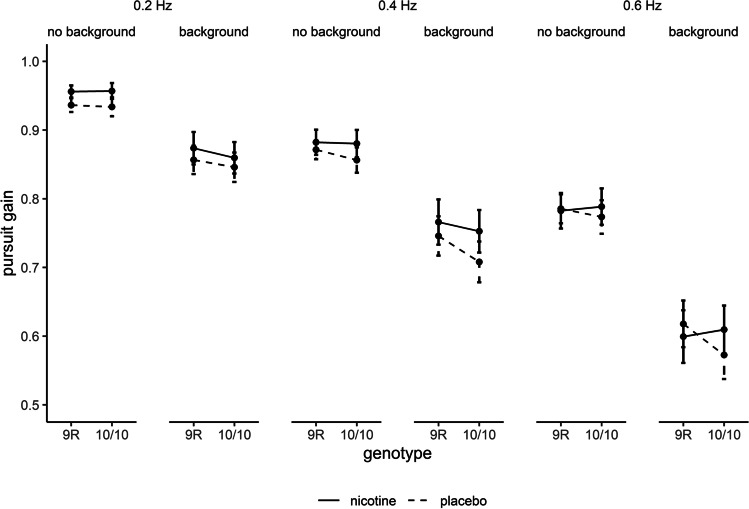
Effects of drug, genotype, target velocity and background on smooth pursuit velocity gain. Data are presented as mean ± standard errors. *N* = 187

Analyses of velocity gain revealed main effects of background (*F*_(1, 183)_ = 286.44, *p* < .001, $\eta$_p_^2^ = .610) and velocity (*F*_(2, 366)_ = 391.69, *p* < .001, $\eta$_p_^2^ = .682, $\epsilon$ = .74) and a two-way interaction of background and target velocity (*F*_(2, 366)_ = 56.85, *p* < .001, $\eta$_p_^2^ = .237, $\epsilon$ = .94; Figure 4). Bonferroni-corrected *t*-tests revealed significant differences between the background conditions at all target velocities (0.2 Hz: background vs. no background *t*_(186)_ = -10.75, *p* < .001, *d*_av_ = -.76; 0.4 Hz: background vs. no background *t*_(186)_ = -13.62, *p* < .001, *d*_av_ = -.78; 0.6 Hz: background vs. no background *t*_(186)_ = -17.77, *p* < .001, *d*_av_ = -.89). However, background effects were larger at higher target velocities. Differences between all target velocity conditions were significant at each background level (background: 0.2 Hz vs. 0.4 Hz *t*_(186)_ = 13.80, *p* < .001, *d*_av_ = .64; background: 0.2 Hz vs. 0.6 Hz *t*_(186)_ = 21.58, *p* < .001, *d*_av_ = 1.31; background: 0.4 Hz vs. 0.6 Hz *t*_(186)_ = 15.27, *p* < .001, *d*_av_ = .63; no background: 0.2 Hz vs. 0.4 Hz *t*_(186)_ = 12.86, *p* < .001, *d*_av_ = .74; no background: 0.2 Hz vs. 0.6 Hz *t*_(186)_ = 17.97, *p* < .001, *d*_av_ = 1.33; no background: 0.4 Hz vs. 0.6 Hz *t*_(186)_ = 13.95, *p* < .001, *d*_av_ = .62). There were no main effects of drug or genotype and no further interactions (all *p* > .05).

Conditional process analysis yielded no significant paths (Supplementary Tables 3 and 4). The index of moderated mediation was 0.015 (SE = 0.472) [-1.296; 0.702]. The bootstrap confidence interval included zero, suggesting a nonsignificant effect.

The Bayesian ANOVA suggested that the data were best represented by a model including the factor target velocity, background and their interaction (see Supplementary Table 5). There was extreme evidence to include the main effects of target velocity (BF_incl_ = 7.460e+12) and background (BF_incl_ = 7.460e+12) as well as their interaction (BF_incl_ = 5.359e+7) and strong evidence to exclude the main effects of drug (BF_excl_ = 18.51) and genotype (BF_excl_ = 21.99) as well as strong to extreme evidence to exclude all other interactions (all BF_excl_ >= 21.74; see Supplementary Table 6).

### Stop signal

In line with assumptions of the race model (Verbruggen et al. 2019), reaction times in go trials were significantly larger than in incorrect stop trials (*t*_(160)_ = 19.20, *p* < .001, *d*_av_ = .68).

**Fig. 5:**
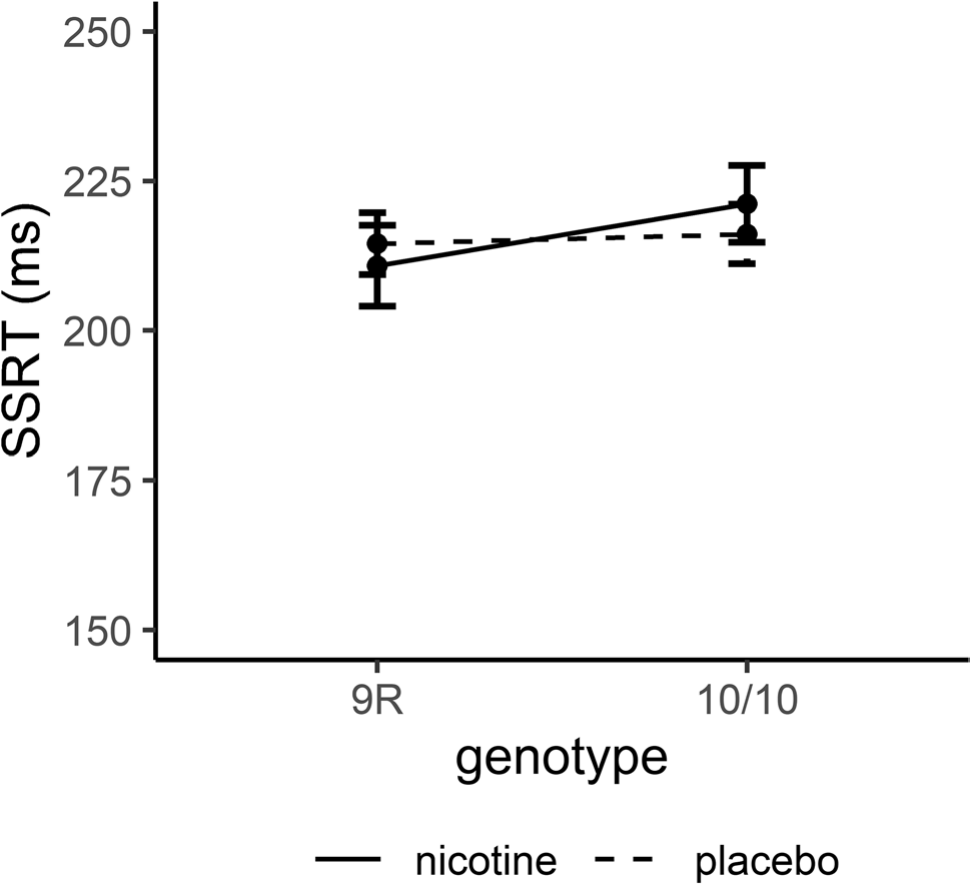
Effects of drug and genotype on stop signal reaction time. Data are presented as mean ± standard errors. SSRT: stop signal reaction time. *N* = 161.

ANOVA on SSRT did not result in any main or interaction effects of drug and genotype (all *p* > .05; Figure 5).

Conditional process analysis yielded no significant paths (Supplementary Tables 7 and 8). The index of moderated mediation was -0.012 (SE = 1.473) [-3.94; 2.234]. The bootstrap confidence interval included zero, suggesting a nonsignificant effect.

The Bayesian ANOVA suggested that the data were best represented by a null model (see Supplementary Table 9). There was moderate evidence to exclude drug (BF_excl_ = 8.21) and genotype (BF_excl_ = 5.15) and strong evidence to exclude their interaction (BF_excl_ = 27.19) (see Supplementary Table 10).

### Proactive inhibition

**Fig. 6:**
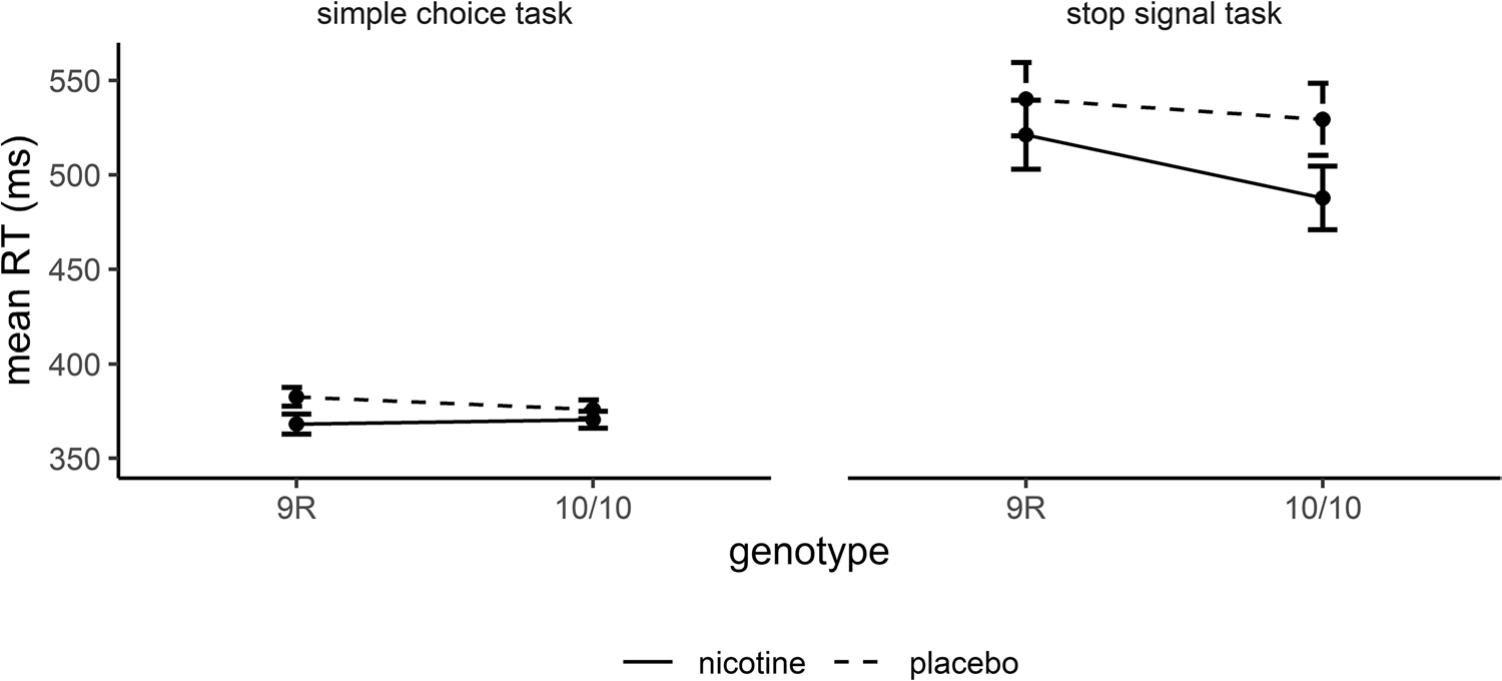
Effects of drug and genotype on go reaction times in the simple choice and stop signal tasks. Data are presented as mean ± standard errors. RT: reaction time. *N* = 182.

ANOVA on go reaction times revealed significant main effects of drug (*F*_(1, 178)_ = 3.90, *p* = .0499, $\eta$_p_^2^ = .021) and task (*F*_(1, 178)_ = 270.45, *p* < .001, $\eta$_p_^2^ = .603; Figure 6). Reaction times were shorter under nicotine vs. placebo and in the simple choice task compared to the stop signal task. There were no interactions and no main effect of genotype (all *p* > .05).

Conditional process analysis yielded no significant paths (Supplementary Tables 11 and 12). The index of moderated mediation was 0.451 (SE = 3.464) [-7.056; 8.097]. The bootstrap confidence interval included zero, suggesting a nonsignificant effect.

The Bayesian ANOVA suggested that the data were best represented by a model including only the factor task (see Supplementary Table 13). There was extreme evidence for the inclusion of task (BF_incl_ = 2.924e+14), anecdotal evidence for the exclusion of drug (BF_excl_ = 2.30) and moderate evidence to exclude genotype (BF_excl_ = 6.65). There was moderate evidence for the exclusion of the task × drug (BF_excl_ = 3.07) and task × genotype (BF_excl_ = 5.67) interactions, strong evidence for the exclusion of drug × genotype (BF_excl_ = 16.43) and extreme evidence for the exclusion of the task × drug × genotype (BF_excl_ = 116.31) interactions (see Supplementary Table 14).

### ACP

**Fig. 7:**
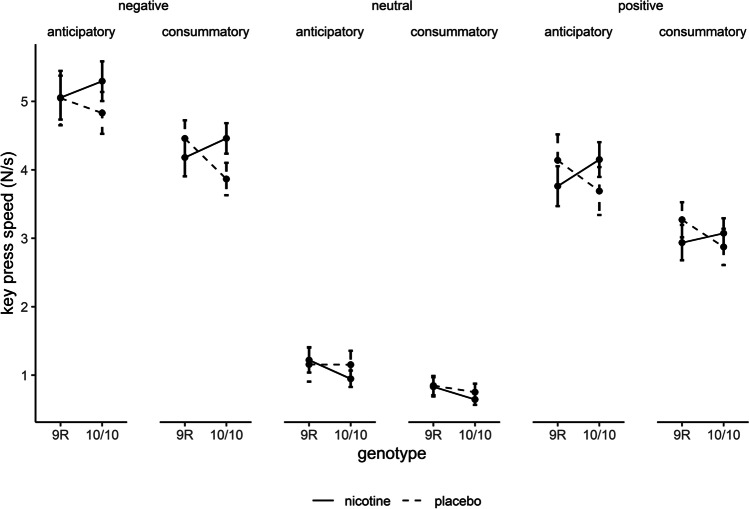
Effects of drug and genotype on key press speed in the ACP task. Data are presented as mean ± standard errors. *N* = 191.

ANOVA on key press speed revealed significant main effects of valence (*F*_(2, 374)_ = 564.36, *p* < .001, $\eta$_p_^2^ = .751, $\epsilon$ = .96) and phase (*F*_(1, 187)_ = 78.53, *p* < .001, $\eta$_p_^2^ = .296; Figure 7). Key press speed was higher for negative and positive compared to neutral slides (negative vs. neutral *t*_(190)_ = 31.00, *p* < .001, *d*_av_ = 2.35; negative vs. positive *t*_(190)_ = 11.60, *p* < .001, *d*_av_ = .57; neutral vs. positive *t*_(190)_ = -22.04, *p* < .001, *d*_av_ = -1.63) and higher for the anticipatory compared to the consummatory phase. In addition, there was a significant interaction of valence and phase (*F*_(2, 374)_ = 15.06, *p* < .001, $\eta$_p_^2^ = .075, $\epsilon$ = .93) suggesting that differences between the two phases were bigger for positive and negative slides than for neutral slides. However, all differences were significant (negative: anticipatory vs. consummatory *t*_(190)_ = 8.22, *p* < .001, *d*_av_ = .41; neutral: anticipatory vs. consummatory *t*_(190)_ = 5.00, *p* < .001, *d*_av_ = .32; positive: anticipatory vs. consummatory *t*_(190)_ = 7.52, *p* < .001, *d*_av_ = .46). There were no further main effects or interactions (all *p* > .05).

Conditional process analysis yielded no significant paths (Supplementary Tables 15 and 16). The index of moderated mediation was 0.092 (SE = 0.072) [-0.128; 0.179]. The bootstrap confidence interval included zero, suggesting a nonsignificant effect.

The Bayesian ANOVA suggested that the data were best represented by a model including the factors phase and valence as well as their interaction (see Supplementary Table 17). There was extreme evidence for the inclusion of valence (BF_incl_ = 9.883e+12) and phase (BF_incl_ = 9.883e+12) main effects and their interaction (BF_incl_ = 7.607e+13). There was very strong to extreme evidence to exclude the main effects of drug and genotype and all other interactions (all BF_excl_ >= 35.27; see Supplementary Table 18).

Descriptive statistics of all task effects are summarized in Supplementary Table 19.

### Cardiovascular effects

Heart rate was higher under nicotine compared to placebo (*t*_(192)_ = 2.53, *p* = .01, *d* = 0.36), but nicotine had no effect on blood pressure (all *p* > .05; Table 3).

**Table 3:** Descriptive statistics of cardiovascular parameters and subjective experience in the nicotine and placebo groups

	Nicotine	Placebo
Heart rate	75.06 (11.58)	71.21 (9.49)
Systolic blood pressure	112.99 (12.33)	110.80 (11.91)
Diastolic blood pressure	74.42 (7.63)	72.34 (7.50)
VAS alertness	59.69 (16.81)	65.82 (16.06)
VAS contentedness	70.37 (14.76)	72.47 (12.15)
VAS calmness	67.66 (20.10)	74.69 (17.32)

Legend: Numbers indicate mean and standard deviations (in brackets). VAS: visual analogue scales. Heart rate is given in contractions per minute, systolic and diastolic blood pressure are given in millimetres of mercury, VAS values are average scale values with higher values indicating higher expression on the factors (range 0-100).

*N* = 194.

### Visual analogue scales

*T*-tests of nicotine influence on subjective feelings revealed significant effects on alertness (*t*_(192)_ = -2.60, *p = .*01, *d* = -0.37) and calmness (*t*_(192)_ = -2.61, *p = .*01, *d* = -0.37), but not on contentedness (*t*_(192)_ = -1.08, *p = .*28, *d* = -0.16). Participants receiving nicotine reported to be less alert and less calm than participants receiving placebo. Table 3 shows the descriptive statistics of heart rate, blood pressure and VAS.

### Individual substance identification

On average, participants guessed correctly which substance they had received (${\chi }_{1}^{2}$ = 18.48, *p* < .001).

## Discussion

Nicotine is a widely used substance with pro-dopaminergic effects. However, studies examining nicotine effects on cognitive, motor and affective functioning in healthy non-smokers yield heterogenous results suggesting substantial between-subject variance, possibly related to genetic variations.

This preregistered study focused on a candidate polymorphism in a gene related to dopaminergic functioning, the *SLC6A3* 3’ UTR VNTR. 194 participants were grouped according to genotype and received either nicotine or placebo in a randomized 2×2-between-subjects design. In order to test whether the assumed nicotine × genotype interactions depend on striatal dopamine activity, SBR was obtained and conditional process analyses testing the mediating effect of SBR on task performance were conducted.

In the frequentist analysis approach, nicotine reduced go reaction times in simple choice and stop signal tasks but had no effect on smooth pursuit performance, reactive and proactive inhibition and affective processing. Bayesian ANOVA, however, revealed anecdotal evidence against nicotine effects on reaction times, but confirmed the null effects on all other tasks. In addition, in frequentist and Bayesian analyses, no interactions with genotype were observed. Conditional process analyses showed no significant results of substance or genotype. Nicotine increased heart rate and decreased subjective ratings of alertness and calmness. Participants guessed above chance-level, which substance they had received.

### Nicotine effects

#### Fixation

Contrary to our hypothesis, no modulatory effect of nicotine on SBR could be observed despite the well-established role of the nicotinic acetylcholine receptor in striatal dopamine functioning and prior evidence of nicotine effects on SBR (Klein et al. 1993; de Kloet et al. 2015). Importantly, in the past, pharmacological enhancement of dopamine functioning has not consistently increased SBR (Jongkees and Colzato 2016). For example, Cavanagh et al. (2014) showed that administration of the dopamine-D2-receptor agonist cabergoline enhanced SBR only in participants with low placebo SBR and decreased it in participants with high placebo SBR. Unfortunately, baseline dependency effects cannot be investigated in our between-subjects study but should be considered in future work.

In a recent study, no relationship between SBR and striatal D2 availability and, more importantly, no drug-related SBR modulation following dopamine agonist bromocriptine administration was observed in healthy participants (Dang et al. 2017). This result along with our null finding suggests that the relationship between SBR and pharmacologically challenged dopamine activity in healthy humans is less straight-forward than expected. Importantly, as suggested by Dang et al. (2017) correlations between SBR and dopamine functioning might only become apparent under more extreme circumstances, such as dopamine-related clinical diseases, but not in healthy participants.

An additional line of argument is that nicotine influences dopamine neurotransmission indirectly via the cholinergic system (de Kloet et al. 2015), but also via activation of γ-aminobutyric acid (GABA) and glutamate (Wonnacott et al. 2005). Consequently, it can be argued that nicotine may not be specific enough to induce the hypothesized effects on SBR.

#### SPEM

In contrast to our previous study (Meyhöfer et al. 2019) we could not find any nicotine effect on SPEM. This result is surprising given the large effect size in Meyhöfer et al. (2019), who used the same paradigm, application protocol and dose. The reason for this rather contradictory result is not entirely clear, but the most striking difference between the studies is the use of between- vs. within-subject drug administration. We addressed this matter by using a much larger sample with sufficient power to detect the effects observed by Meyhöfer et al. (2019), but still failed to replicate them in our study.

It is worth noting that other studies too have been unsuccessful in showing improved SPEM performance in response to nicotine in healthy non-smokers (Kasparbauer et al. 2016; Olincy et al. 1998; Sibony et al. 1988). Crucially, however, results are more consistent in groups with impaired dopamine neurotransmission, such as patients with schizophrenia. Here, mostly beneficial effects of nicotine have been reported (Olincy et al. 1998; Sherr et al. 2002; Tregellas et al. 2005). This suggests that nicotine may positively influence SPEM only when performance is at a suboptimal level.

Unfortunately, our between-subjects approach does not allow analysis of baseline dependent drug effects that have been revealed in the past for other oculomotor tasks (Babin et al. 2011). Low-dose nicotine effects on SPEM might be so subtle that they only emerge in participants with lower-than-average performance or in study designs with better control of interindividual variability.

#### Reactive inhibition

Beneficial effects of nicotine on SSRT have been found in samples with lower-than-average inhibition performance such as deprived smokers or highly impulsive individuals (Potter and Newhouse 2004; Tsaur et al. 2015). In healthy non-smokers, however, results are less consistent. While one study found weak effects (Logemann et al. 2014b), others have failed to do so (Ettinger et al. 2017; Logemann et al. 2014a; Wignall and de Wit 2011). Our results are in line with the latter, confirming that nicotine does not show inhibition enhancing effects in the stop signal task in healthy, non-smoking individuals.

#### Proactive inhibition

The only task-related measure where nicotine effects were found in frequentist analyses, were go reaction times in the stop signal and simple choice tasks. Reaction times were significantly reduced by nicotine, indicative of enhanced motor responses under nicotine, consistent with meta-analytic findings of improved fine motor performance and decreased alerting attention reaction times under nicotine (Heishman et al. 2010). This finding may, at least in part, be attributed to nicotinic action at peripheral, striatal and motor cortex sites (Dani and Bertrand 2007; Heishman et al. 2010; Mansvelder et al. 2006). Importantly, however, Bayesian analyses provided anecdotal (or inconclusive) evidence against nicotine effects on reaction times. This points to the need for more research to better understand the exact effects of nicotine on reaction times.

Nicotine did not differentially affect reaction times in the stop signal and simple choice tasks, suggesting no specific drug effect on proactive inhibition.

#### ACP

Nicotine did not affect key press speed in the ACP task implying that affective processing is not altered by drug administration. This is in contrast to previous investigations describing nicotine-induced effects on incentive motivation, reward processing and affective responses to film clips (Barr et al. 2008a; Dawkins et al. 2006; Dawkins and Powell 2011). Our data suggest that motivated behaviour to alter probability or presentation times of emotional stimulus displays is unaffected by nicotine administration. In contrast to the simple choice and stop signal tasks, no general facilitation of motor responses could be observed. This may be the results of differences in task instructions. While participants were instructed to react as fast and accurately as possible with a single key press to the stimuli in the simple choice and stop signal tasks, instructions in the ACP required sustained responses over a period of several seconds as a result of in-depth processing of the stimulus and evaluation of one’s own motivational state. Thus, simple reaction times are the result of fast visuomotor transformations as a consequence of simple stimulus-response associations while ACP reactions reflect more complex, self-generated effortful behaviour in anticipation of or in direct response to an affective stimulus.

### Genotype

Overall, our results show that nicotine might facilitate motor responses, as indicated by the decrease in reaction times in the simple choice and stop signal tasks which – of note – was not confirmed in Bayesian analyses but has no effects on other outcomes. Importantly, considering the level of genetics (*SLC6A3* 3’ UTR VNTR 9R-carriers vs. 10R-homozygotes) with a view to tapping differences in DAT-related dopamine neurotransmission did not help to explain interindividual differences in nicotine response in healthy non-smokers and thus cannot contribute to resolving inconsistencies in the literature. While this is in contrast to the majority of previous investigations (Brewer et al. 2015; Franklin et al. 2009, 2011; Gelernter et al. 1994; Millar et al. 2011), it is not the first study that failed to observe differences in response to pro-dopaminergic pharmacological manipulations between the genotype groups (Hart et al. 2013; Kambeitz et al. 2014). Accordingly, previous findings might reflect false positive results of poorly-powered studies (Hart et al. 2013) or only apply to specific substances or tasks.

Of note, DAT availability is not static and can change, e.g. with smoking status or drug administration (Newberg et al. 2007; Schmitt and Reith, 2010; Yang et al. 2008). Here, we only included healthy, young non-smokers who did not regularly consume other drugs, while smoking and drug administration status might have been less well controlled in previous investigations

### Task effects

#### SPEM

SPEM velocity gain decreased with higher target velocities and in the presence of a structured background. In addition, we showed a significant interaction between the target velocity and background factors, indicating that negative background effects are larger at higher target velocities. These results are in excellent agreement with our earlier investigations employing the same paradigm, closely replicating previously observed effect sizes (Meyhöfer et al. 2019; Schröder et al. 2021). Increasing task demands due to higher velocity targets or stationary background could challenge processes inherent to pursuit performance, such as spatial attention and motion perception (Kerzel et al. 2008; Ohlendorf et al. 2010), leading to the observed decreases in velocity gain. Along with the recent finding of high test-retest and split-half reliability in this task (Schröder et al. 2021) the replication of these task effects at group level underlines the robustness of this paradigm for both the current and future investigations of SPEM.

#### Stop signal

The race model underlying stop signal task performance is based on the assumption that go and stop responses are triggered by the presentation of go and stop stimuli, respectively. The first of these two processes to finish, determines whether a response is executed or stopped (Logan and Cowan 1984). The SSRT is a measure of the duration of the stop process (Logan and Cowan 1984). In the present study, the stop signal task was carried out in accordance with recommendations of a recently published consensus guide (Verbruggen et al. 2019). To make sure that race model assumptions were met, we excluded participants according to strict criteria (described in "Stop signal task"). Moreover, reaction times in go trials were higher than in incorrect stop trials, confirming the independence of the stop and go processes. The high number of excluded participants was possibly due to the relatively small step size (16 ms) in the tracking procedure combined with only 50 stop trials. However, after exclusion, the sample size was still so large, that drug effects of medium size could have been detected. Taken together, it may be concluded that stop signal reaction time was validly assessed in this study although no evidence for a modulation by nicotine could be uncovered.

#### Proactive inhibition

In agreement with the literature, reaction times to go stimuli were significantly higher in the stop signal task than the simple choice task (Chikazoe et al. 2009; Verbruggen and Logan 2009; Vink et al. 2015). This pattern of results suggests that participants proactively slowed their responses in the stop signal task in anticipation of the requirement to inhibit the motor response. This is probably achieved by adjusting the individual response strategy by trading speed in the simple choice task for success in the stop signal task despite the explicit instruction not to wait for the stop signal (Verbruggen and Logan 2009). Although the increase in response time in the stop signal task can be the result of two different processes, proactive adjustment and dual-task requirements, the former is considered to play a larger role (Verbruggen and Logan 2009). Overall, our results confirm the validity of the approach to compare tasks with and without stop trials in order to study proactive inhibition although no nicotine effect could be observed.

#### ACP

So far, the ACP task has mainly been used to study clinical groups, such as patients with schizophrenia, where decreased coupling of subjective experience and behaviour was observed (Heerey and Gold 2007; Lui et al. 2016). In the present study, we replicated valence and phase effects indicating increased key press speed to negative and positive compared to neutral slides and in the anticipatory compared to the consummatory phase. The fact that participants engaged in more effortful behaviour in anticipation than in direct response to an emotional stimulus display is in line with the notion that behavioural responses are more tightly linked to wanting than to liking of stimuli (Berridge 2007; Pool et al. 2016). Key press speed was higher to negative than positive slides, indicative of negativity bias in affective processing (Ito et al. 1998).

The replicability of these task effects indicates that the ACP is an adequate method for quantifying motivational behaviour.

### Cardiovascular and subjective effects

The finding of increased heart rate with nicotine (Benowitz et al. 1982, 1988; Logemann et al. 2014a) is likely due to excitatory nicotinic effects on the sympathetic nervous system (Adamopoulos et al. 2008). Significant effects on other cardiovascular measures could not be observed, possibly due to the low dose applied here.

At the level of subjective experience, we observed negative nicotine effects on alertness and calmness, but no effect on contentedness. The calmness effect is consistent with a meta-analysis suggesting decreased subjective relaxation levels after nicotine administration in both smokers and non-smokers (Kalman and Smith 2005). Similarly, the effect on alertness matches the decreased vigour ratings found under nicotine in the same meta-analysis (Kalman and Smith 2005). In light of the attention-enhancing effects of nicotine (Hahn 2015; Heishman et al. 2010), decreased alertness ratings seem surprising at first sight. However, negative subjective effects along with decreased reaction times have also been reported in previous studies (Ettinger et al. 2017; Heishman and Henningfield, 2000). Importantly, subjective ratings of alertness covered a broad range of feelings compared to the relatively specific measure of reaction times in response to a stimulus following task instructions. This may suggest that in non-smokers objective improvements in performance are overshadowed by negative subjective nicotine effects or that nicotine can improve some aspects of cognition without the concomitant subjective experience of this improvement. This may lead to the conclusion that subjective and behavioural outcomes are differentially affected by nicotine. It should also be noted, however, that some studies did not observe any subjective nicotine effects (Meyhöfer et al. 2019; Thiel and Fink 2007) or effects opposite to ours (Griesar et al. 2002; Warburton and Mancuso 1998). The heterogeneity in subjective nicotine effects might be attributed to different approaches in assessing subjective experience, dosage, smoking status and baseline subjective state (Griesar et al. 2002; Kalman and Smith 2005; Perkins et al. 1992).

### General limitations

Our results should be considered in the light of some limitations.

First, we did not collect baseline data on SBR prior to drug administration. Therefore, potential baseline dependency effects could not be explored (Jongkees and Colzato 2016; Unsworth et al. 2019). This may be critical as there is substantial variability in SBR (Unsworth et al. 2019) and nicotine administration might exert non-linear effects (Cavanagh et al. 2014; Cools and D’Esposito 2011) that could not be captured with our approach. Similarly, baseline data for other dependent variables such as task performance and subjective feelings may also have helped to qualify the observed results (Ettinger et al. 2017; Perkins et al. 1992).

Second, every participant received the same nicotine dose and nicotine plasma levels were not monitored. However, we tried to control nicotine intake as accurately as possible by presenting a standardized chewing protocol and we restricted the sample to participants with normal body weight according to BMI criteria.

Third, a 2 mg nicotine dose might have been too low to induce the expected effects. Notably, however, participants were selected according to strict non-smoking criteria and the same dose has yielded positive effects on a broad range of outcomes in non-smokers in the past (Almeida et al. 2020; Meinke et al. 2006; Meyhöfer et al. 2019). Also, even with this relatively low dose, two participants had to discontinue their study participation because of adverse side effects. Presumably, this number would have been even higher with higher dosage (Nyberg et al. 1982).

Fourth, we did not directly measure dopamine turnover via single photon emission computed tomography (SPECT) or positron emission tomography (PET) due to the invasiveness and costs of such methods. Instead, we decided to rely on indirect measures of striatal dopamine activity, i.e. SBR, that might have been too imprecise for our purposes.

### Conclusion

To conclude, across a number of a priori selected oculomotor, cognitive and affective outcomes, beneficial effects of nicotine were observed in a large sample of healthy non-smokers only for reaction times to go stimuli in stop signal and simple choice tasks. Of note, this effect was small and not supported – but also not conclusively ruled out - by Bayesian analyses. Against our preregistered hypothesis - but confirmed by Bayesian analyses - *SLC6A3* 3’ UTR VNTR genotype (9R-carriers and 10R-homozygotes) did not interact with nicotine administration. SBR as a measure of striatal dopamine activity was not affected by nicotine and unrelated to performance. Nicotine had negative effects on subjective ratings on alertness and calmness and increased heart rate, in accordance with previous investigations. Taken together, our results highlight the need for more well-powered research to characterize the association between dopaminergic genes and response to pharmacological challenges.

## Supplementary Information

Below is the link to the electronic supplementary material.

Supplementary file1 (PDF 677 KB)
