# Supplementary material for: The role of the SLC6A3 3’ UTR VNTR in nicotine effects on cognitive, affective, and motor function
Source: Psychopharmacology (Berl). 2021 Dec 2;239(2):489–507. doi: 10.1007/s00213-021-06028-x (PMC8638222; doi:10.1007/s00213-021-06028-x)
Supplement: Supplementary file 1 — Supplementary file1 (PDF 677 KB) [file 213_2021_6028_MOESM1_ESM.pdf]

SUPPLEMENTARY MATERIALS:

The Role of the *SLC6A3* 3’ UTR VNTR in Nicotine Effects on Cognitive, Affective and Motor Function

Rebekka Schröder[1], Martin Reuter[1], Kaja Faßbender[1], Thomas Plieger[1], Jessie Poulsen[2], Simon S. Y. Lui[3], Raymond C. K. Chan[4,5], Ulrich Ettinger[1*]

[1] *Department of Psychology, University of Bonn, Bonn, Germany*

[2] *Nicotine Science Center, Fertin Pharma A/S, Vejle, Denmark*

[3] *Department of Psychiatry, The University of Hong Kong, Hong Kong Special Administrative Region, China*

[4] *Neuropsychology and Applied Cognitive Neuroscience (NACN) Laboratory, CAS Key Laboratory of Mental Health, Institute of Psychology, Beijing, China*

[5] *Department of Psychology, University of Chinese Academy of Sciences, Beijing, China*

*** Corresponding author:**

Ulrich Ettinger, Department of Psychology, University of Bonn, Kaiser-Karl-Ring 9, 53111 Bonn, Germany

Email: ulrich.ettinger@uni-bonn.de, Phone: +49 228 734208

Supplementary Figure 1: Smooth pursuit target display

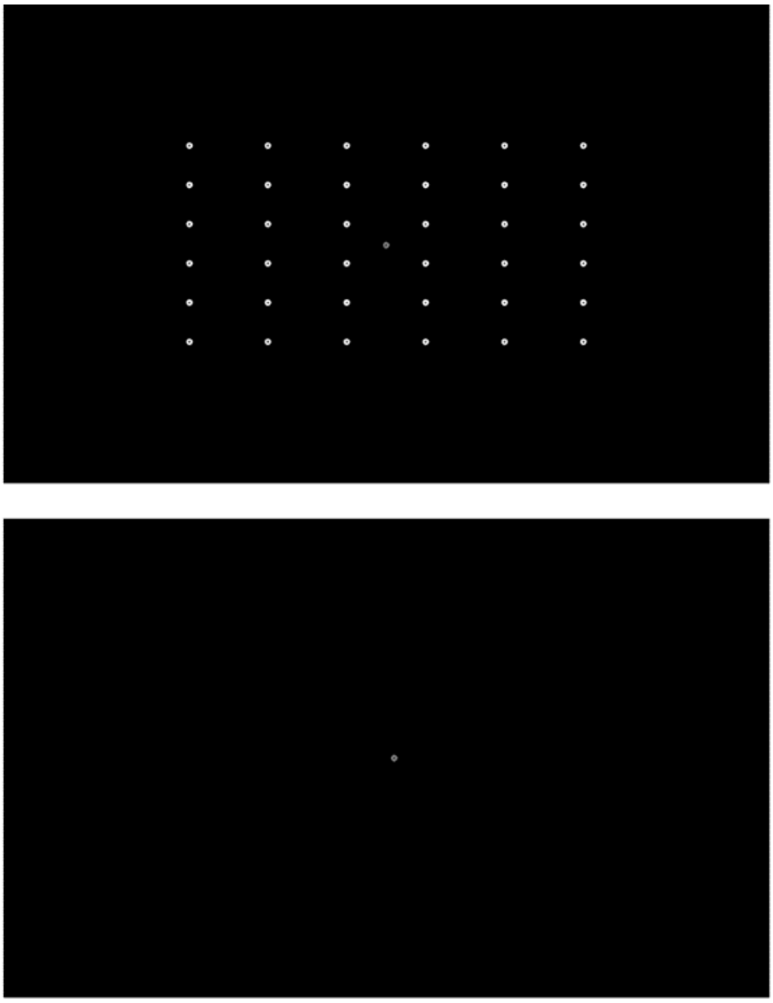

Legend: The upper panel depicts the target display in the background condition. The lower panel depicts the target display in the no background condition. Both panels show the grey target at the centre of the screen.

Supplementary Figure 2: Schematic illustration of the trial sequence in the stop signal task

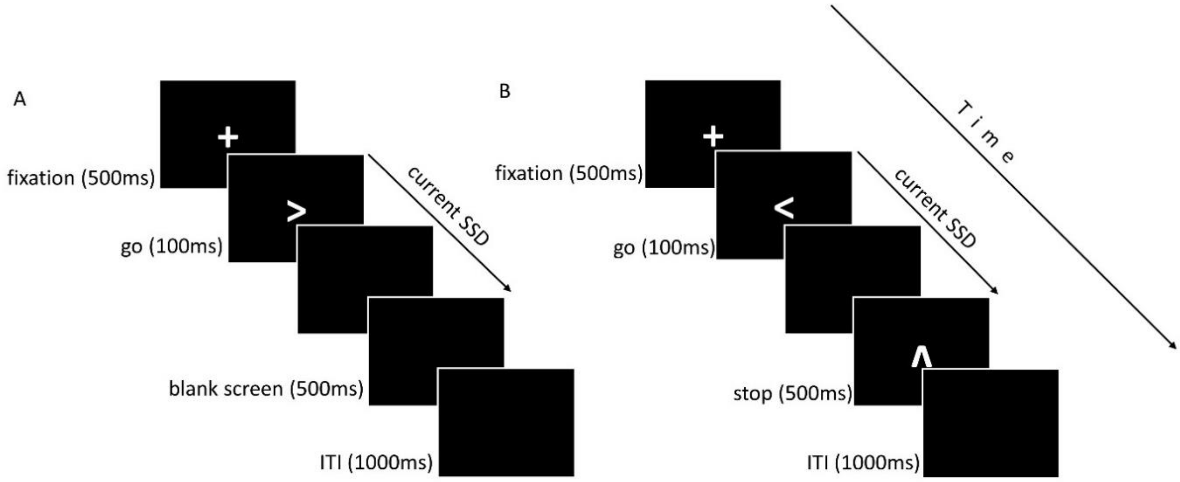

Legend: Trial sequence in a go trial (panel A) and a stop trial (panel B) in the stop signal task. ITI = intertrial interval, SSD = stop signal delay.

Supplementary Figure 3: Schematic illustration of the trial sequence in the simple choice task

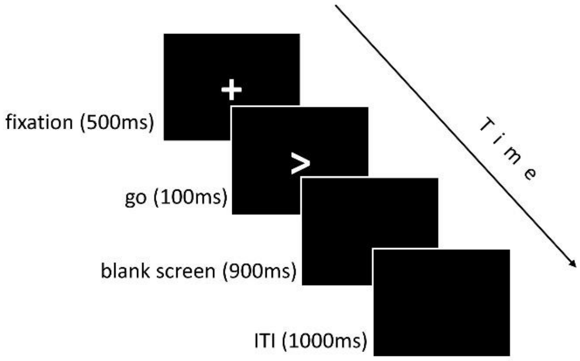

Legend: Trial sequence in the simple choice task. ITI = intertrial interval.

Supplementary Figure 4: Schematic illustration of the trial sequence in the ACP

A

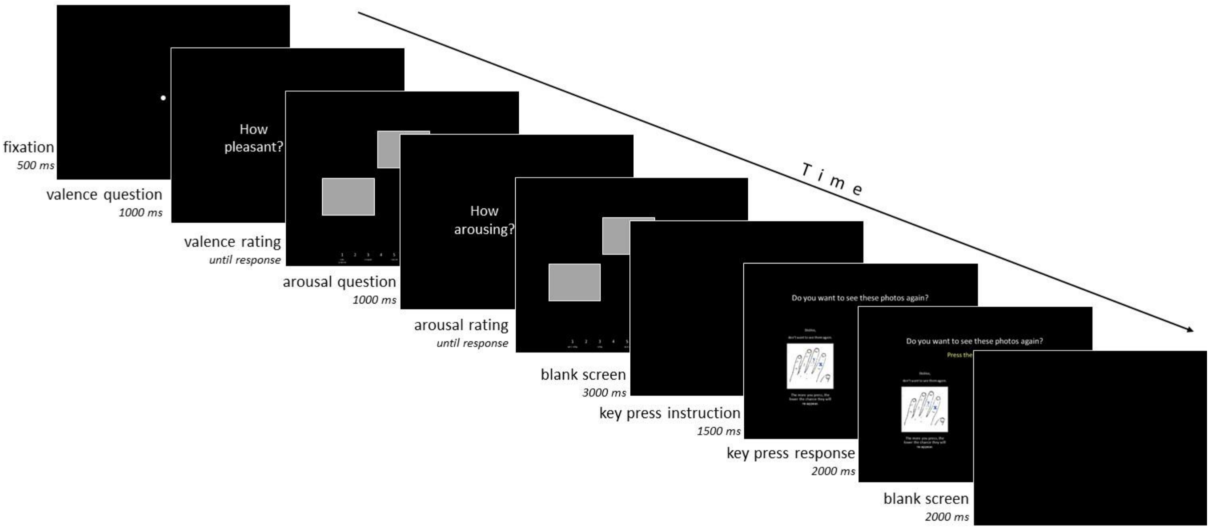

B

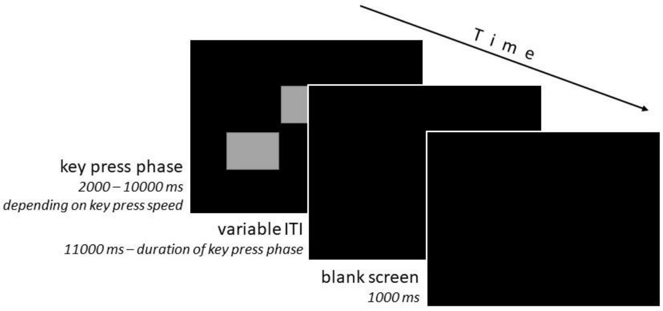

Legend: Panel A: Trial sequence in the first (anticipatory) phase of the ACP task. Panel B: Trial sequence in the second (consummatory) phase of the ACP task. The grey rectangles serve as representation for the IAPS images not depicted here due to copyright restrictions.

Spontaneous blink rate

Supplementary Table 1: Results of Bayesian model comparison for spontaneous blink rate

Models	P(M)	P(M|data)	BF_M	BF_{01}	error %
Null model	0.200	0.712	9.871	1.000	
genotype	0.200	0.136	0.629	5.234	6.833e-6
drug	0.200	0.124	0.566	5.740	7.009e-6
drug + genotype	0.200	0.024	0.097	29.975	1.122
drug + genotype + drug × genotype	0.200	0.005	0.019	151.505	2.338

Legend: P(M): prior model probability, P(M|data): posterior model probability, BF_M: posterior model odds, BF_{01}: evidence for the best model (first row) relative to alternative model, error %: error of the Gaussian quadrature integration routine used for the computation of the Bayes factor.

Supplementary Table 2: Analysis of effects of Bayesian ANOVA for spontaneous blink rate

Effects	P(incl)	P(excl)	P(incl|data)	P(excl|data)	BF_{excl}
drug	0.600	0.400	0.152	0.848	8.342
genotype	0.600	0.400	0.164	0.836	7.624
drug × genotype	0.200	0.800	0.005	0.995	52.975

Legend: P(incl): prior inclusion probability, P(excl): prior exclusion probability, P(incl|data): posterior inclusion probability, P(excl|data): posterior exclusion probability, BF_{excl}: exclusion Bayes factor.

Smooth pursuit gain

Supplementary Table 3: Regression model coefficients for the effects of drug and genotype on spontaneous eye blink rate and SPEM velocity gain

	Consequent							
	M (SBR)				Y (SPEM velocity gain)			
Model summary	$R^2 = 0.00$<br>$F_{(3,183)} = 0.10$<br>$p = .963$				$R^2 = 0.01$<br>$F_{(4,182)} = 0.56$<br>$p = .695$			
Antecedent		Coeff.	*SE*	*p*		Coeff.	*SE*	*p*
Constant	i_M	0.17	0.03	< .001	i_Y	79.38	2.50	< .001
X (drug)	a_1	0.01	0.03	.83	c'_1	2.68	3.01	.38
M (SBR)		---	---	---	b	-7.09	6.46	.27
W (genotype)	a_2	-0.01	0.04	.78	c'_2	1.99	3.09	.52
X × W	a_3	-0.00	0.05	.97	c'_3	-1.87	4.33	.67

Legend: Results of the conditional process analysis of the effects of drug and genotype on smooth pursuit velocity gain, mediated by spontaneous eye blink rate. SBR: spontaneous eye blink rate; *SE:* standard error; Coeff.: coefficient. X = antecedent; M = mediator; W = moderator; X × W = interaction between mediator and moderator.

Supplementary Table 4: Conditional direct and indirect effects of drug on SPEM velocity gain

	Direct Effect				Indirect Effect			
Genotype	Coeff.	*SE*	Confidence interval		Coeff.	*SE*	Bootstrap confidence interval	
			LB	UB			LB	UB
10/10	2.68	3.01	-3.26	8.62	-0.05	0.35	-0.73	0.83
9R	0.81	3.11	-5.33	6.95	-0.04	0.34	-0.96	0.44

Legend: Conditional direct and indirect effects of drug on SPEM velocity gain for the two genotype groups. *SE*: standard error; Coeff.: coefficient; LB: lower bound of confidence interval; UB: upper bound of confidence interval.

Supplementary Table 5: Results of Bayesian model comparison for smooth pursuit gain

Models	P(M)	P(M|data)	BF_M	BF_{01}	error %
velocity + background + velocity × background	0.006	0.522	181.498	1.000	
velocity + background + drug + velocity × background	0.006	0.194	40.028	2.688	16.574
velocity + background + genotype + velocity × background	0.006	0.147	28.573	3.557	4.948
velocity + background + drug + genotype + velocity × background	0.006	0.042	7.212	12.544	3.655
velocity + background + genotype + velocity × background + background × genotype	0.006	0.031	5.278	16.949	11.056
velocity + background + drug + genotype + velocity × background + drug × genotype	0.006	0.016	2.659	33.133	11.158
velocity + background + drug + velocity × background + background × drug	0.006	0.015	2.534	34.740	5.061
velocity + background + drug + genotype + velocity × background + background × genotype	0.006	0.008	1.298	67.299	4.665
velocity + background + drug + velocity × background + velocity × drug	0.006	0.006	1.036	84.240	4.172
velocity + background + drug + genotype + velocity × background + background × drug	0.006	0.004	0.635	136.969	3.953
velocity + background + drug + genotype + velocity × background + background × genotype + drug × genotype	0.006	0.003	0.553	157.313	16.550
velocity + background + genotype + velocity × background + velocity × genotype	0.006	0.003	0.541	160.845	4.015
velocity + background + drug + genotype + velocity × background + velocity × drug	0.006	0.002	0.290	299.987	7.366
velocity + background + drug + genotype + velocity × background + background × drug + drug × genotype	0.006	0.001	0.237	366.697	14.035
velocity + background + drug + genotype + velocity × background + velocity × genotype	0.006	0.001	0.194	446.938	15.359
velocity + background + drug + genotype + velocity × background + background × drug + background × genotype	0.006	8.403e-4	0.140	621.598	8.080
velocity + background + drug + genotype + velocity × background + velocity × drug + drug × genotype	0.006	6.661e-4	0.111	784.144	27.531
velocity + background + genotype + velocity × background + velocity × genotype + background × genotype	0.006	6.146e-4	0.102	849.828	3.709
velocity + background + drug + velocity × background + velocity × drug + background × drug	0.006	5.761e-4	0.096	906.585	3.841
velocity + background + drug + genotype + velocity × background + velocity × genotype + drug × genotype	0.006	3.096e-4	0.051	1687.091	19.366
velocity + background + drug + genotype + velocity × background + velocity × drug + background × genotype	0.006	3.026e-4	0.050	1725.760	4.420
velocity + background + drug + genotype + velocity × background + background × drug + background × genotype + drug × genotype	0.006	2.929e-4	0.049	1782.984	14.598
velocity + background + drug + genotype + velocity × background + velocity × genotype + background × genotype	0.006	2.016e-4	0.033	2590.764	3.951
velocity + background + drug + genotype + velocity × background + velocity × drug + background × drug	0.006	1.943e-4	0.032	2688.642	23.657
velocity + background + drug + genotype + velocity × background + background × drug + velocity × genotype	0.006	1.008e-4	0.017	5182.026	7.694
velocity + background + drug + genotype + velocity × background + velocity × drug + background × genotype + drug × genotype	0.006	9.395e-5	0.016	5559.294	21.795
velocity + background + drug + genotype + velocity × background + velocity × genotype + background × genotype + drug × genotype	0.006	6.956e-5	0.012	7508.532	30.685

Models	P(M)	P(M|data)	BF_M	BF_{01}	error %
velocity + background + drug + genotype + velocity × background + velocity × drug + background × drug + drug × genotype	0.006	6.796e-5	0.011	7685.280	18.471
velocity + background + drug + genotype + velocity × background + velocity × drug + velocity × genotype	0.006	5.308e-5	0.009	9840.379	16.598
velocity + background + drug + genotype + velocity × background + background × drug + background × genotype + drug × genotype + background × drug × genotype	0.006	3.161e-5	0.005	16523.284	15.954
velocity + background + genotype + velocity × background + velocity × genotype + background × genotype + velocity × background × genotype	0.006	3.066e-5	0.005	17035.536	25.095
velocity + background + drug + velocity × background + velocity × drug + background × drug + velocity × background × drug	0.006	2.949e-5	0.005	17712.257	4.926
velocity + background + drug + genotype + velocity × background + velocity × drug + background × drug + background × genotype	0.006	2.742e-5	0.005	19045.625	4.210
velocity + background + drug + genotype + velocity × background + background × drug + velocity × genotype + drug × genotype	0.006	2.689e-5	0.004	19420.249	17.421
velocity + background + drug + genotype + velocity × background + background × drug + velocity × genotype + background × genotype	0.006	2.027e-5	0.003	25764.503	6.123
velocity + background + drug + genotype + velocity × background + velocity × drug + background × drug + background × genotype + drug × genotype	0.006	1.808e-5	0.003	28880.920	31.567
velocity + background + drug + genotype + velocity × background + velocity × drug + velocity × genotype + drug × genotype	0.006	1.456e-5	0.002	35870.352	16.373
velocity + background + drug + genotype + velocity × background + background × drug + velocity × genotype + background × genotype + drug × genotype	0.006	9.488e-6	0.002	55051.193	20.328
velocity + background + drug + genotype + velocity × background + velocity × drug + velocity × genotype + background × genotype	0.006	8.364e-6	0.001	62443.118	9.148
velocity + background + drug + genotype + velocity × background + velocity × genotype + background × genotype + velocity × background × genotype	0.006	7.963e-6	0.001	65591.508	6.832
velocity + background + drug + genotype + velocity × background + velocity × drug + background × drug + velocity × background × drug	0.006	7.691e-6	0.001	67914.434	5.464
velocity + background + drug + genotype + velocity × background + velocity × drug + background × drug + velocity × genotype	0.006	4.002e-6	6.644e-4	130493.892	5.962
velocity + background + drug + genotype + velocity × background + velocity × drug + background × drug + drug × genotype + velocity × background × drug	0.006	3.818e-6	6.339e-4	136784.633	35.883
velocity + background + drug + genotype + velocity × background + velocity × drug + velocity × genotype + background × genotype + drug × genotype	0.006	2.581e-6	4.285e-4	202350.298	19.654
velocity + background + drug + genotype + velocity × background + velocity × genotype + background × genotype + drug × genotype + velocity × background × genotype	0.006	2.172e-6	3.605e-4	240503.371	21.299

Models	P(M)	P(M|data)	BF_M	BF_{01}	error %
velocity + background + drug + genotype + velocity × background + velocity × drug + background × drug + background × genotype + drug × genotype + background × drug × genotype	0.006	1.838e-6	3.051e-4	284175.214	27.321
velocity + background + drug + genotype + velocity × background + velocity × drug + background × drug + background × genotype + velocity × background × drug	0.006	1.329e-6	2.206e-4	393078.134	18.830
velocity + background + drug + genotype + velocity × background + velocity × drug + background × drug + velocity × genotype + drug × genotype	0.006	1.150e-6	1.909e-4	454226.769	22.422
velocity + background + drug + genotype + velocity × background + background × drug + velocity × genotype + background × genotype + drug × genotype + background × drug × genotype	0.006	9.993e-7	1.659e-4	522654.448	21.713
velocity + background + drug + genotype + velocity × background + velocity × drug + background × drug + velocity × genotype + background × genotype	0.006	8.321e-7	1.381e-4	627686.167	6.310
velocity + background + drug + genotype + velocity × background + background × drug + velocity × genotype + background × genotype + velocity × background × genotype	0.006	8.096e-7	1.344e-4	645126.903	7.894
velocity + background + drug + genotype + velocity × background + velocity × drug + velocity × genotype + drug × genotype + velocity × drug × genotype	0.006	5.833e-7	9.683e-5	895376.286	19.385
velocity + background + drug + genotype + velocity × background + velocity × drug + velocity × genotype + background × genotype + velocity × background × genotype	0.006	3.094e-7	5.136e-5	1.688e+6	4.922
velocity + background + drug + genotype + velocity × background + velocity × drug + background × drug + background × genotype + drug × genotype + velocity × background × drug	0.006	2.931e-7	4.865e-5	1.782e+6	21.215
velocity + background + drug + genotype + velocity × background + background × drug + velocity × genotype + background × genotype + drug × genotype + velocity × background × genotype	0.006	2.047e-7	3.398e-5	2.551e+6	20.613
velocity + background + drug + genotype + velocity × background + velocity × drug + background × drug + velocity × genotype + velocity × background × drug	0.006	1.964e-7	3.260e-5	2.659e+6	10.228
velocity + background + drug + genotype + velocity × background + velocity × drug + background × drug + velocity × genotype + background × genotype + drug × genotype	0.006	1.594e-7	2.646e-5	3.277e+6	16.506
velocity + background + drug + genotype + velocity × background + velocity × drug + velocity × genotype + background × genotype + drug × genotype + velocity × background × genotype	0.006	1.441e-7	2.392e-5	3.625e+6	28.970
velocity + background + drug + genotype + velocity × background + velocity × drug + velocity × genotype + background × genotype + drug × genotype + velocity × drug × genotype	0.006	1.422e-7	2.360e-5	3.673e+6	20.238
velocity + background + drug + genotype + velocity × background + velocity × drug + background × drug + background × genotype + drug × genotype + velocity × background × drug + background × drug × genotype	0.006	7.390e-8	1.227e-5	7.068e+6	40.339
velocity + background + drug + genotype + velocity × background + velocity × drug + background × drug + velocity × genotype + drug × genotype + velocity × drug × genotype	0.006	6.796e-8	1.128e-5	7.686e+6	18.496
velocity + background + drug + genotype + velocity × background + velocity × drug + background × drug + velocity × genotype + background × genotype + drug × genotype + background × drug × genotype	0.006	6.381e-8	1.059e-5	8.185e+6	30.129

Models	P(M)	P(M|data)	BF_M	BF_{01}	error %
velocity + background + drug + genotype + velocity × background + velocity × drug + background × drug + velocity × genotype + drug × genotype + velocity × background × drug	0.006	5.508e-8	9.143e-6	9.483e+6	39.831
velocity + background + drug + genotype + velocity × background + velocity × drug + background × drug + velocity × genotype + background × genotype + velocity × background × drug	0.006	4.710e-8	7.819e-6	1.109e+7	17.714
velocity + background + drug + genotype + velocity × background + background × drug + velocity × genotype + background × genotype + drug × genotype + velocity × background × genotype + background × drug × genotype	0.006	3.210e-8	5.329e-6	1.627e+7	25.561
velocity + background + drug + genotype + velocity × background + velocity × drug + background × drug + velocity × genotype + background × genotype + velocity × background × genotype	0.006	3.180e-8	5.279e-6	1.642e+7	9.166
velocity + background + drug + genotype + velocity × background + velocity × drug + background × drug + velocity × genotype + background × genotype + drug × genotype + velocity × drug × genotype	0.006	2.306e-8	3.828e-6	2.265e+7	36.690
velocity + background + drug + genotype + velocity × background + velocity × drug + background × drug + velocity × genotype + background × genotype + drug × genotype + velocity × background × drug	0.006	1.464e-8	2.431e-6	3.567e+7	25.688
velocity + background	0.006	1.046e-8	1.737e-6	4.991e+7	2.803
velocity + background + drug + genotype + velocity × background + velocity × drug + velocity × genotype + background × genotype + drug × genotype + velocity × background × genotype + velocity × drug × genotype	0.006	6.240e-9	1.036e-6	8.371e+7	33.437
velocity + background + drug + genotype + velocity × background + velocity × drug + background × drug + velocity × genotype + background × genotype + drug × genotype + velocity × background × genotype	0.006	5.420e-9	8.998e-7	9.636e+7	31.626
velocity + background + drug + genotype + velocity × background + velocity × drug + background × drug + velocity × genotype + drug × genotype + velocity × background × drug + velocity × drug × genotype	0.006	3.370e-9	5.593e-7	1.550e+8	33.905
velocity + background + drug	0.006	2.985e-9	4.955e-7	1.750e+8	3.038
velocity + background + genotype	0.006	2.554e-9	4.240e-7	2.045e+8	2.983
velocity + background + drug + genotype + velocity × background + velocity × drug + background × drug + velocity × genotype + background × genotype + drug × genotype + velocity × drug × genotype + background × drug × genotype	0.006	2.188e-9	3.633e-7	2.387e+8	30.206
velocity + background + drug + genotype + velocity × background + velocity × drug + background × drug + velocity × genotype + background × genotype + drug × genotype + velocity × background × drug + background × drug × genotype	0.006	2.023e-9	3.359e-7	2.582e+8	34.700
velocity + background + drug + genotype + velocity × background + velocity × drug + background × drug + velocity × genotype + background × genotype + drug × genotype + velocity × background × drug + velocity × drug × genotype	0.006	1.960e-9	3.254e-7	2.665e+8	59.350
velocity + background + drug + genotype + velocity × background + velocity × drug + background × drug + velocity × genotype + background × genotype + drug × genotype + velocity × background × genotype + background × drug × genotype	0.006	1.826e-9	3.032e-7	2.860e+8	32.173

Models	P(M)	P(M|data)	BF_M	BF_{01}	error %
velocity + background + drug + genotype + velocity × background + velocity × drug + background × drug + velocity × genotype + background × genotype + velocity × background × drug + velocity × background × genotype	0.006	1.546e-9	2.566e-7	3.379e+8	8.424
velocity + background + drug + genotype + velocity × background + velocity × drug + background × drug + velocity × genotype + background × genotype + drug × genotype + velocity × background × genotype + velocity × drug × genotype	0.006	8.607e-10	1.429e-7	6.068e+8	52.641
velocity + background + drug + genotype	0.006	7.684e-10	1.276e-7	6.797e+8	2.960
velocity + background + genotype + background × genotype	0.006	4.976e-10	8.260e-8	1.050e+9	3.397
velocity + background + drug + genotype + velocity × background + velocity × drug + background × drug + velocity × genotype + background × genotype + drug × genotype + velocity × background × drug + velocity × background × genotype	0.006	2.895e-10	4.806e-8	1.804e+9	26.341
velocity + background + drug + background × drug	0.006	2.850e-10	4.731e-8	1.833e+9	2.854
velocity + background + drug + genotype + drug × genotype	0.006	2.357e-10	3.913e-8	2.216e+9	9.547
velocity + background + drug + genotype + velocity × background + velocity × drug + background × drug + velocity × genotype + background × genotype + drug × genotype + velocity × background × genotype + velocity × drug × genotype + background × drug × genotype	0.006	1.965e-10	3.262e-8	2.658e+9	45.929
velocity + background + drug + genotype + background × genotype	0.006	1.915e-10	3.178e-8	2.728e+9	21.158
velocity + background + drug + velocity × drug	0.006	1.169e-10	1.940e-8	4.468e+9	2.894
velocity + background + drug + genotype + background × drug	0.006	7.607e-11	1.263e-8	6.866e+9	3.961
velocity + background + drug + genotype + velocity × background + velocity × drug + background × drug + velocity × genotype + background × genotype + drug × genotype + velocity × background × drug + velocity × drug × genotype + background × drug × genotype	0.006	6.866e-11	1.140e-8	7.607e+9	30.521
velocity + background + genotype + velocity × genotype	0.006	5.958e-11	9.891e-9	8.766e+9	2.625
velocity + background + drug + genotype + background × genotype + drug × genotype	0.006	4.322e-11	7.174e-9	1.209e+10	9.623
velocity + background + drug + genotype + velocity × background + velocity × drug + background × drug + velocity × genotype + background × genotype + drug × genotype + velocity × background × drug + velocity × background × genotype + velocity × drug × genotype + background × drug × genotype	0.006	3.820e-11	6.341e-9	1.367e+10	97.083
velocity + background + drug + genotype + velocity × drug	0.006	2.983e-11	4.951e-9	1.751e+10	3.385
velocity + background + drug + genotype + background × drug + drug × genotype	0.006	2.960e-11	4.913e-9	1.765e+10	11.978
velocity + background + drug + genotype + velocity × background + velocity × drug + background × drug + velocity × genotype + background × genotype + drug × genotype + velocity × background × drug + velocity × background × genotype + velocity × drug × genotype	0.006	2.763e-11	4.587e-9	1.890e+10	45.548
velocity + background + drug + genotype + velocity × genotype	0.006	1.856e-11	3.081e-9	2.814e+10	3.002

Models	P(M)	P(M|data)	BF_M	BF_{01}	error %
velocity + background + drug + genotype + velocity × background + velocity × drug + background × drug + velocity × genotype + background × genotype + drug × genotype + velocity × background × drug + velocity × background × genotype + background × drug × genotype	0.006	1.761e-11	2.922e-9	2.967e+10	22.039
velocity + background + drug + genotype + background × drug + background × genotype	0.006	1.407e-11	2.336e-9	3.711e+10	4.688
velocity + background + drug + genotype + velocity × drug + drug × genotype	0.006	1.269e-11	2.107e-9	4.116e+10	23.777
velocity + background + genotype + velocity × genotype + background × genotype	0.006	1.148e-11	1.906e-9	4.548e+10	3.341
velocity + background + drug + velocity × drug + background × drug	0.006	1.084e-11	1.799e-9	4.819e+10	3.441
velocity + background + drug + genotype + velocity × drug + background × genotype	0.006	5.636e-12	9.356e-10	9.267e+10	8.182
velocity + background + drug + genotype + velocity × genotype + drug × genotype	0.006	5.465e-12	9.072e-10	9.557e+10	11.445
velocity + background + drug + genotype + background × drug + background × genotype + drug × genotype	0.006	4.843e-12	8.039e-10	1.079e+11	13.541
velocity + background + drug + genotype + velocity × genotype + background × genotype	0.006	3.555e-12	5.901e-10	1.469e+11	5.500
velocity + background + drug + genotype + velocity × drug + background × drug	0.006	2.761e-12	4.583e-10	1.892e+11	4.182
velocity + background + drug + genotype + background × drug + velocity × genotype	0.006	1.994e-12	3.310e-10	2.619e+11	5.933
velocity + background + drug + genotype + velocity × drug + background × genotype + drug × genotype	0.006	1.829e-12	3.037e-10	2.855e+11	15.899
velocity + background + drug + genotype + velocity × genotype + background × genotype + drug × genotype	0.006	1.022e-12	1.697e-10	5.110e+11	12.756
velocity + background + drug + genotype + velocity × drug + background × drug + drug × genotype	0.006	8.362e-13	1.388e-10	6.246e+11	22.867
velocity + background + drug + genotype + velocity × drug + velocity × genotype	0.006	8.024e-13	1.332e-10	6.510e+11	8.217
velocity + background + drug + genotype + background × drug + background × genotype + drug × genotype + background × drug × genotype	0.006	7.077e-13	1.175e-10	7.380e+11	16.444
velocity + background + drug + genotype + background × drug + velocity × genotype + drug × genotype	0.006	6.127e-13	1.017e-10	8.525e+11	11.895
velocity + background + drug + genotype + velocity × drug + background × drug + background × genotype	0.006	5.978e-13	9.923e-11	8.738e+11	15.323
velocity + background + drug + genotype + background × drug + velocity × genotype + background × genotype	0.006	3.404e-13	5.651e-11	1.534e+12	3.734
velocity + background + drug + genotype + velocity × drug + velocity × genotype + drug × genotype	0.006	2.151e-13	3.571e-11	2.428e+12	23.776
velocity + background + drug + genotype + velocity × drug + velocity × genotype + background × genotype	0.006	1.375e-13	2.283e-11	3.798e+12	4.429
velocity + background + drug + genotype + background × drug + velocity × genotype + background × genotype + drug × genotype	0.006	1.129e-13	1.874e-11	4.628e+12	32.369
velocity + background + drug + genotype + velocity × drug + background × drug + background × genotype + drug × genotype	0.006	1.082e-13	1.796e-11	4.827e+12	13.565
velocity + background + drug + genotype + velocity × background + velocity × drug + background × drug + velocity × genotype + background × genotype + drug × genotype + velocity × background × drug + velocity × background × genotype + velocity × drug × genotype + background × drug × genotype + velocity × background × drug × genotype	0.006	9.419e-14	1.563e-11	5.545e+12	34.131
velocity + background + drug + genotype + velocity × drug + background × drug + velocity × genotype	0.006	7.942e-14	1.318e-11	6.577e+12	7.840

Models	P(M)	P(M|data)	BF_M	BF_{01}	error %
velocity + background + drug + genotype + velocity × drug + background × drug + velocity × genotype + drug × genotype	0.006	3.922e-14	6.510e-12	1.332e+13	35.339
velocity + background + drug + genotype + velocity × drug + velocity × genotype + background × genotype + drug × genotype	0.006	3.799e-14	6.307e-12	1.375e+13	21.528
velocity + background + drug + genotype + velocity × drug + background × drug + background × genotype + drug × genotype + background × drug × genotype	0.006	3.492e-14	5.797e-12	1.496e+13	23.162
velocity + background + drug + genotype + background × drug + velocity × genotype + background × genotype + drug × genotype + background × drug × genotype	0.006	2.518e-14	4.180e-12	2.074e+13	25.388
velocity + background + drug + genotype + velocity × drug + velocity × genotype + drug × genotype + velocity × drug × genotype	0.006	1.888e-14	3.135e-12	2.766e+13	17.822
velocity + background + drug + genotype + velocity × drug + background × drug + velocity × genotype + background × genotype	0.006	1.239e-14	2.057e-12	4.216e+13	3.630
velocity + background + drug + genotype + velocity × drug + velocity × genotype + background × genotype + drug × genotype + velocity × drug × genotype	0.006	3.684e-15	6.115e-13	1.418e+14	22.737
velocity + background + drug + genotype + velocity × drug + background × drug + velocity × genotype + background × genotype + drug × genotype	0.006	2.582e-15	4.285e-13	2.023e+14	22.653
velocity + background + drug + genotype + velocity × drug + background × drug + velocity × genotype + drug × genotype + velocity × drug × genotype	0.006	1.061e-15	1.761e-13	4.922e+14	25.163
velocity + background + drug + genotype + velocity × drug + background × drug + velocity × genotype + background × genotype + drug × genotype + background × drug × genotype	0.006	9.882e-16	1.640e-13	5.285e+14	43.362
velocity + background + drug + genotype + velocity × drug + background × drug + velocity × genotype + background × genotype + drug × genotype + velocity × drug × genotype	0.006	5.382e-16	8.934e-14	9.705e+14	41.309
velocity + background + drug + genotype + velocity × drug + background × drug + velocity × genotype + background × genotype + drug × genotype + velocity × drug × genotype + background × drug × genotype	0.006	5.429e-17	9.013e-15	9.620e+15	28.938
velocity	0.006	1.195e-96	1.984e-94	4.371e+95	2.032
velocity + drug	0.006	2.891e-97	4.800e-95	1.806e+96	2.700
velocity + genotype	0.006	2.598e-97	4.313e-95	2.010e+96	4.219
velocity + drug + genotype	0.006	6.045e-98	1.003e-95	8.641e+96	2.315
velocity + drug + genotype + drug × genotype	0.006	1.711e-98	2.840e-96	3.053e+97	2.507
velocity + drug + velocity × drug	0.006	8.481e-99	1.408e-96	6.158e+97	2.196
velocity + genotype + velocity × genotype	0.006	5.615e-99	9.321e-97	9.301e+97	2.270
velocity + drug + genotype + velocity × drug	0.006	1.825e-99	3.030e-97	2.862e+98	2.350
velocity + drug + genotype + velocity × genotype	0.006	1.457e-99	2.418e-97	3.586e+98	2.901
velocity + drug + genotype + velocity × drug + drug × genotype	0.006	5.684e-100	9.436e-98	9.188e+98	5.432
velocity + drug + genotype + velocity × genotype + drug × genotype	0.006	3.780e-100	6.275e-98	1.382e+99	2.591

Models	P(M)	P(M|data)	BF_M	BF_{01}	error %
velocity + drug + genotype + velocity × drug + velocity × genotype	0.006	4.353e-101	7.225e-99	1.200e+100	2.872
velocity + drug + genotype + velocity × drug + velocity × genotype + drug × genotype	0.006	1.324e-101	2.198e-99	3.944e+100	4.233
velocity + drug + genotype + velocity × drug + velocity × genotype + drug × genotype + velocity × drug × genotype	0.006	7.786e-103	1.292e-100	6.709e+101	4.780
background	0.006	2.555e-139	4.241e-137	2.044e+138	2.171
background + drug	0.006	5.765e-140	9.570e-138	9.059e+138	2.792
background + genotype	0.006	4.800e-140	7.968e-138	1.088e+139	2.610
background + drug + genotype	0.006	1.080e-140	1.793e-138	4.835e+139	2.619
background + genotype + background × genotype	0.006	6.168e-141	1.024e-138	8.469e+139	2.854
background + drug + background × drug	0.006	5.235e-141	8.690e-139	9.977e+139	3.109
background + drug + genotype + drug × genotype	0.006	2.675e-141	4.440e-139	1.953e+140	2.601
background + drug + genotype + background × genotype	0.006	1.400e-141	2.324e-139	3.731e+140	3.085
background + drug + genotype + background × drug	0.006	9.566e-142	1.588e-139	5.460e+140	6.227
background + drug + genotype + background × genotype + drug × genotype	0.006	3.757e-142	6.236e-140	1.390e+141	5.091
background + drug + genotype + background × drug + drug × genotype	0.006	2.754e-142	4.571e-140	1.897e+141	5.734
background + drug + genotype + background × drug + background × genotype	0.006	1.447e-142	2.403e-140	3.609e+141	8.298
background + drug + genotype + background × drug + background × genotype + drug × genotype	0.006	3.579e-143	5.941e-141	1.459e+142	5.801
background + drug + genotype + background × drug + background × genotype + drug × genotype + background × drug × genotype	0.006	5.353e-144	8.886e-142	9.758e+142	8.634
Null model (incl. subject)	0.006	4.473e-189	7.426e-187	1.168e+188	1.855
drug	0.006	8.814e-190	1.463e-187	5.925e+188	2.042
genotype	0.006	7.288e-190	1.210e-187	7.167e+188	1.988
drug + genotype	0.006	1.458e-190	2.420e-188	3.583e+189	2.328
drug + genotype + drug × genotype	0.006	3.390e-191	5.627e-189	1.541e+190	2.513

Legend: P(M): prior model probability, P(M|data): posterior model probability, BF_M: posterior model odds, BF_{01}: evidence for the best model (first row) relative to alternative model, error %: error of the Gaussian quadrature integration routine used for the computation of the Bayes factor.

Supplementary Table 6: Analysis of effects of Bayesian ANOVA for smooth pursuit gain

Effects	P(incl)	P(excl)	P(incl|data)	P(excl|data)	BF_{excl}
velocity	0.886	0.114	1.000	1.721e-14	1.340e-13
background	0.886	0.114	1.000	1.721e-14	1.340e-13
drug	0.886	0.114	0.296	0.704	18.514
genotype	0.886	0.114	0.262	0.738	21.990
velocity × background	0.503	0.497	1.000	1.844e-8	1.866e-8
velocity × drug	0.503	0.497	0.010	0.990	100.004
velocity × genotype	0.503	0.497	0.006	0.994	170.583
background × drug	0.503	0.497	0.023	0.977	43.905
background × genotype	0.503	0.497	0.044	0.956	21.742
drug × genotype	0.503	0.497	0.022	0.978	44.734
velocity × background × drug	0.120	0.880	4.302e-5	1.000	3162.795
velocity × background × genotype	0.120	0.880	4.234e-5	1.000	3213.073
velocity × drug × genotype	0.120	0.880	8.315e-7	1.000	163629.978
background × drug × genotype	0.120	0.880	3.462e-5	1.000	3929.418
velocity × background × drug × genotype	0.006	0.994	9.419e-14	1.000	6.396e +10

Legend: P(incl): prior inclusion probability, P(excl): prior exclusion probability, P(incl|data): posterior inclusion probability, P(excl|data): posterior exclusion probability, BF_{excl}: exclusion Bayes factor.

Stop signal reaction time

Supplementary Table 7: Regression model coefficients for the effects of drug and genotype on spontaneous eye blink rate and SSRT

		Consequent						
		M (SBR)				Y (SSRT)		
Model summary		$R^2 = 0.01$, $F_{(3,156)} = 0.44$, p =.73				$R^2 = 0.02$, $F_{(4,155)} = 0.64$, p =.63		
Antecedent		Coeff.	*SE*	*p*		Coeff.	*SE*	*p*
Constant	i_M	0.16	0.02	< .001	i_Y	213.64	7.09	< .001
X (drug)	a_1	-0.00	0.03	.89	c'_1	4.35	8.41	.61
M (SBR)		---	---	---	b	21.14	22.09	.34
W (genotype)	a_2	0.01	0.03	.84	c'_2	-2.55	8.60	.77
X × W	a_3	-0.03	0.04	.50	c'_3	-7.34	11.97	.54

Legend: Results of the conditional process analysis of the effects of drug and genotype on SSRT (stop signal reaction time), mediated by spontaneous eye blink rate. SBR: spontaneous eye blink rate; *SE:* standard error; Coeff.: coefficient. X = antecedent; M = mediator; W = moderator; X × W = interaction between mediator and moderator.

Supplementary Table 8: Conditional direct and indirect effects of drug on SSRT

	Direct Effect				Indirect Effect			
Genotype	Coeff.	*SE*	Confidence interval		Coeff.	*SE*	Bootstrap confidence interval	
			LB	UB			LB	UB
10/10	4.35	8.41	-12.28	20.97	-0.09	1.01	-2.46	1.86
9R	-2.99	8.52	-19.82	13.84	-0.70	1.14	-3.43	1.23

Legend: Conditional direct and indirect effects of drug on SSRT (stop signal reaction time) for the two genotype groups. *SE:* standard error; Coeff.: coefficient; LB: lower bound of confidence interval; UB: upper bound of confidence interval.

Supplementary Table 9: Results of Bayesian model comparison for stop signal reaction time

Models	P(M)	P(M|data)	BF_M	BF_{01}	error %
Null model	0.200	0.660	7.779	1.000	
genotype	0.200	0.185	0.909	3.567	6.937e-6
drug	0.200	0.114	0.514	5.798	1.044e-5
drug + genotype	0.200	0.031	0.130	21.014	1.105
drug + genotype + drug × genotype	0.200	0.009	0.037	72.480	2.849

Legend: P(M): prior model probability, P(M|data): posterior model probability, BF_M: posterior model odds, BF_{01}: evidence for the best model (first row) relative to alternative model, error %: error of the Gaussian quadrature integration routine used for the computation of the Bayes factor.

Supplementary Table 10: Analysis of effects of Bayesian ANOVA for stop signal reaction time

Effects	P(incl)	P(excl)	P(incl|data)	P(excl|data)	BF_{excl}
drug	0.600	0.400	0.154	0.846	8.212
genotype	0.600	0.400	0.226	0.774	5.147
drug × genotype	0.200	0.800	0.009	0.991	27.187

Legend: P(incl): prior inclusion probability, P(excl): prior exclusion probability, P(incl|data): posterior inclusion probability, P(excl|data): posterior exclusion probability, BF_{excl}: exclusion Bayes factor.

Go reaction times

Supplementary Table 11: Regression model coefficients for the effects of drug and genotype on spontaneous eye blink rate and go reaction time difference between stop and simple choice tasks

	Consequent							
	M (SBR)				Y (go reaction time difference)			
Model summary	$R^2 = 0.00$<br>$F_{(3,177)} = 0.05$<br>$p = .98$				$R^2 = 0.02$<br>$F_{(4,176)} = 0.94$<br>$p = .44$			
Antecedent		Coeff.	*SE*	*p*		Coeff.	*SE*	*p*
Constant	i_M	0.17	0.02	<.001	i_Y	148.13	20.68	< .001
X (drug)	a_1	-0.01	0.03	.71	c'_1	-35.76	-24.80	.15
M (SBR)		---	---	---	b	30.45	55.94	.59
W (genotype)	a_2	-0.01	0.03	.77	c'_2	4.40	25.29	.86
X × W	a_3	0.01	0.05	.76	c'_3	26.40	35.31	.46

Legend: Results of the conditional process analysis of the effects of drug and genotype on go reaction time difference between the stop and simple choice tasks, mediated by spontaneous eye blink rate. SBR: spontaneous eye blink rate; *SE:* standard error; Coeff.: coefficient. X = antecedent; M = mediator; W = moderator; X × W = interaction between mediator and moderator.

Supplementary Table 12: Conditional direct and indirect effects of drug on go reaction time difference between stop and simple choice tasks

	Direct Effect				Indirect Effect			
Genotype	Coeff.	*SE*	Confidence interval		Coeff.	*SE*	Bootstrap confidence interval	
			LB	UB			LB	UB
10/10	-35.76	24.80	-84.71	13.19	-0.38	2.58	-6.33	4.82
9R	-9.36	25.13	-58.95	40.23	0.07	2.25	-5.41	4.69

Legend: Conditional direct and indirect effects of drug on go reaction time difference between stop and simple choice tasks for the two genotype groups. *SE:* standard error; Coeff.: coefficient; LB: lower bound of confidence interval; UB: upper bound of confidence interval.

Supplementary Table 13: Results of Bayesian model comparison for go reaction times in stop and simple choice tasks

Models	P(M)	P(M|data)	BF_M	BF_{01}	error %
task	0.053	0.324	8.614	1.000	
task + drug	0.053	0.290	7.353	1.116	1.880
task + genotype	0.053	0.094	1.860	3.456	1.664
task + drug + task × drug	0.053	0.090	1.779	3.598	2.396
task + drug + genotype	0.053	0.082	1.607	3.950	8.290
task + genotype + task × genotype	0.053	0.033	0.623	9.674	3.874
task + drug + genotype + task × genotype	0.053	0.027	0.492	12.155	5.036
task + drug + genotype + task × drug	0.053	0.025	0.470	12.718	3.850
task + drug + genotype + drug × genotype	0.053	0.015	0.276	21.397	6.765
task + drug + genotype + task × drug + task × genotype	0.053	0.008	0.144	40.710	5.011
task + drug + genotype + task × drug + drug × genotype	0.053	0.005	0.090	65.004	4.047
task + drug + genotype + task × genotype + drug × genotype	0.053	0.005	0.088	66.523	2.762
task + drug + genotype + task × drug + task × genotype + drug × genotype	0.053	0.002	0.034	173.017	18.854
task + drug + genotype + task × drug + task × genotype + drug × genotype + task × drug × genotype	0.053	4.774e-4	0.009	677.936	4.381
Null model (incl. subject)	0.053	2.697e-42	4.854e-41	1.200e +41	1.002
drug	0.053	1.189e-42	2.141e-41	2.721e +41	3.065
genotype	0.053	5.620e-43	1.012e-41	5.759e +41	1.664
drug + genotype	0.053	2.264e-43	4.076e-42	1.429e +42	2.704
drug + genotype + drug × genotype	0.053	3.911e-44	7.040e-43	8.275e +42	2.174

Legend: P(M): prior model probability, P(M|data): posterior model probability, BF_M: posterior model odds, BF_{01}: evidence for the best model (first row) relative to alternative model, error %: error of the Gaussian quadrature integration routine used for the computation of the Bayes factor.

Supplementary Table 14: Analysis of effects of Bayesian ANOVA for go reaction times in stop and simple choice tasks

Effects	P(incl)	P(excl)	P(incl|data)	P(excl|data)	BF_{excl}
task	0.737	0.263	1.000	1.221e-15	3.419e-15
drug	0.737	0.263	0.549	0.451	2.298
genotype	0.737	0.263	0.296	0.704	6.647
task × drug	0.316	0.684	0.131	0.869	3.070
task × genotype	0.316	0.684	0.075	0.925	5.672
drug × genotype	0.316	0.684	0.027	0.973	16.433
task × drug × genotype	0.053	0.947	4.774e-4	1.000	116.313

Legend: P(incl): prior inclusion probability, P(excl): prior exclusion probability, P(incl|data): posterior inclusion probability, P(excl|data): posterior exclusion probability, BF_{excl}: exclusion Bayes factor.

ACP key press speed

Supplementary Table 15: Regression model coefficients for the effects of drug and genotype on spontaneous eye blink rate and ACP key press speed

	Consequent							
	M (SBR)				Y (key press speed)			
Model summary	$R^2 = 0.00$<br>$F_{(3,185)} = .17$<br>$p = .91$				$R^2 = 0.03$<br>$F_{(4,184)} = 1.21$<br>$p = .31$			
Antecedent		Coeff.	*SE*	*p*		Coeff.	*SE*	*p*
Constant	i_M	0.17	0.03	< .001	i_Y	4.07	0.29	< .001
X (drug)	a_1	0.02	0.03	.65	c'_1	0.42	0.35	.23
M (SBR)		---	---	---	b	-1.31	0.75	.08
W (genotype)	a_2	-0.01	0.04	.88	c'_2	0.37	0.36	.30
X × W	a_3	-0.01	0.05	.76	c'_3	-0.63	0.50	.21

Legend: Results of the conditional process analysis of the effects of drug and genotype on key press speed in the ACP task, mediated by spontaneous eye blink rate. SBR: spontaneous eye blink rate; *SE:* standard error; Coeff.: coefficient. X = antecedent; M = mediator; W = moderator; X × W = interaction between mediator and moderator.

Supplementary Table 16: Conditional direct and indirect effects of drug on ACP key press speed

	Direct Effect				Indirect Effect			
Genotype	Coeff.	*SE*	Confidence interval		Coeff.	*SE*	Bootstrap confidence interval	
			LB	UB			LB	UB
10/10	0.42	0.35	-0.27	1.20	-0.02	0.05	-0.15	0.08
9R	-0.22	0.36	-0.92	0.49	-0.00	0.05	-0.10	0.10

Legend: Conditional direct and indirect effects of drug on key press speed in the ACP task for the two genotype groups. *SE*: standard error; Coeff.: coefficient; LB: lower bound of confidence interval; UB: upper bound of confidence interval.

Supplementary Table 17: Results of Bayesian model comparison for ACP key press speed

Models	P(M)	P(M|data)	BF_M	BF_{01}	error %
phase + valence + phase × valence	0.006	0.688	366.227	1.000	
phase + valence + genotype + phase × valence	0.006	0.125	23.788	5.490	4.637
phase + valence + drug + phase × valence	0.006	0.114	21.429	6.018	4.167
phase + valence + drug + genotype + phase × valence	0.006	0.021	3.489	33.423	3.971
phase + valence + genotype + phase × valence + phase × genotype	0.006	0.014	2.419	47.916	4.850
phase + valence + drug + phase × valence + phase × drug	0.006	0.012	1.976	58.483	5.064
phase + valence + drug + genotype + phase × valence + drug × genotype	0.006	0.008	1.272	90.472	5.345
phase + valence + drug + phase × valence + valence × drug	0.006	0.004	0.737	155.738	5.686
phase + valence + genotype + phase × valence + valence × genotype	0.006	0.004	0.604	189.738	22.054
phase + valence + drug + genotype + phase × valence + phase × genotype	0.006	0.002	0.410	279.586	4.723
phase + valence + drug + genotype + phase × valence + phase × drug	0.006	0.002	0.361	317.232	4.195
phase + valence + drug + genotype + phase × valence + phase × genotype + drug × genotype	0.006	9.071e-4	0.151	758.579	8.345
phase + valence + drug + genotype + phase × valence + phase × drug + drug × genotype	0.006	7.948e-4	0.132	865.757	6.318
phase + valence + drug + genotype + phase × valence + valence × drug	0.006	7.786e-4	0.129	883.762	5.035
phase + valence + drug + genotype + phase × valence + valence × genotype	0.006	4.747e-4	0.079	1449.482	4.347
phase + valence + drug + phase × valence + phase × drug + valence × drug	0.006	4.352e-4	0.072	1581.137	5.075
phase + valence + genotype + phase × valence + phase × genotype + valence × genotype	0.006	3.266e-4	0.054	2106.988	4.913
phase + valence + drug + genotype + phase × valence + valence × drug + drug × genotype	0.006	3.034e-4	0.050	2267.607	7.751
phase + valence + drug + genotype + phase × valence + phase × drug + phase × genotype + drug × genotype	0.006	2.335e-4	0.039	2946.339	56.452
phase + valence + drug + genotype + phase × valence + phase × drug + phase × genotype	0.006	2.293e-4	0.038	3000.885	4.555
phase + valence + drug + genotype + phase × valence + valence × genotype + drug × genotype	0.006	1.764e-4	0.029	3901.311	5.116
phase + valence + drug + genotype + phase × valence + valence × drug + phase × genotype	0.006	8.983e-5	0.015	7660.430	4.811
phase + valence + drug + genotype + phase × valence + phase × drug + valence × drug	0.006	7.823e-5	0.013	8795.421	4.983
phase + valence + drug + genotype + phase × valence + phase × genotype + valence × genotype + drug × genotype	0.006	6.620e-5	0.011	10393.666	70.407
phase + valence + drug + genotype + phase × valence + phase × genotype + valence × genotype	0.006	5.706e-5	0.009	12059.119	5.177
phase + valence + drug + genotype + phase × valence + phase × drug + valence × genotype	0.006	5.159e-5	0.009	13337.408	6.389
phase + valence + drug + genotype + phase × valence + valence × drug + phase × genotype + drug × genotype	0.006	3.793e-5	0.006	18140.108	23.193

Models	P(M)	P(M|data)	BF_M	BF_{01}	error %
phase + valence + drug + genotype + phase × valence + phase × drug + valence × drug + drug × genotype	0.006	3.058e-5	0.005	22502.569	6.789
phase + valence + drug + phase × valence + phase × drug + valence × drug + phase × valence × drug	0.006	3.021e-5	0.005	22774.560	4.580
phase + valence + genotype + phase × valence + phase × genotype + valence × genotype + phase × valence × genotype	0.006	1.971e-5	0.003	34906.301	27.746
phase + valence + drug + genotype + phase × valence + valence × drug + valence × genotype	0.006	1.762e-5	0.003	39047.249	7.579
phase + valence + drug + genotype + phase × valence + phase × drug + valence × genotype + drug × genotype	0.006	1.696e-5	0.003	40580.559	5.324
phase + valence + drug + genotype + phase × valence + valence × drug + valence × genotype + drug × genotype + valence × drug × genotype	0.006	1.303e-5	0.002	52827.812	7.133
phase + valence + drug + genotype + phase × valence + phase × drug + phase × genotype + drug × genotype + phase × drug × genotype	0.006	1.113e-5	0.002	61847.931	4.975
phase + valence + drug + genotype + phase × valence + phase × drug + valence × drug + phase × genotype	0.006	8.471e-6	0.001	81227.142	5.823
phase + valence + drug + genotype + phase × valence + valence × drug + valence × genotype + drug × genotype	0.006	6.607e-6	0.001	104146.732	6.671
phase + valence + drug + genotype + phase × valence + phase × drug + valence × drug + phase × valence × drug	0.006	6.238e-6	0.001	110303.084	6.173
phase + valence + drug + genotype + phase × valence + phase × drug + phase × genotype + valence × genotype	0.006	6.152e-6	0.001	111844.892	9.016
phase + valence + drug + genotype + phase × valence + phase × drug + valence × drug + phase × genotype + drug × genotype	0.006	4.401e-6	7.306e-4	156350.918	27.320
phase + valence + drug + genotype + phase × valence + valence × drug + phase × genotype + valence × genotype	0.006	2.798e-6	4.645e-4	245912.062	29.832
phase + valence + drug + genotype + phase × valence + phase × drug + valence × drug + drug × genotype + phase × valence × drug	0.006	2.692e-6	4.469e-4	255607.122	12.257
phase + valence + drug + genotype + phase × valence + phase × genotype + valence × genotype + phase × valence × genotype	0.006	2.250e-6	3.735e-4	305841.826	5.739
phase + valence + drug + genotype + phase × valence + phase × drug + valence × drug + valence × genotype	0.006	2.100e-6	3.485e-4	327738.309	13.519
phase + valence + drug + genotype + phase × valence + phase × drug + phase × genotype + valence × genotype + drug × genotype	0.006	1.963e-6	3.259e-4	350503.885	9.579
phase + valence + drug + genotype + phase × valence + valence × drug + phase × genotype + valence × genotype + drug × genotype + valence × drug × genotype	0.006	1.340e-6	2.224e-4	513566.077	5.970
phase + valence + drug + genotype + phase × valence + phase × drug + valence × drug + valence × genotype + drug × genotype + valence × drug × genotype	0.006	1.206e-6	2.002e-4	570471.198	6.591

Models	P(M)	P(M|data)	BF_M	BF_{01}	error %
phase + valence + drug + genotype + phase × valence + phase × genotype + valence × genotype + drug × genotype + phase × valence × genotype	0.006	7.902e-7	1.312e-4	870810.289	7.284
phase + valence + drug + genotype + phase × valence + valence × drug + phase × genotype + valence × genotype + drug × genotype	0.006	6.905e-7	1.146e-4	996538.501	5.209
phase + valence + drug + genotype + phase × valence + phase × drug + valence × drug + phase × genotype + phase × valence × drug	0.006	6.671e-7	1.107e-4	1.032e+6	7.105
phase + valence + drug + genotype + phase × valence + phase × drug + valence × drug + valence × genotype + drug × genotype	0.006	6.313e-7	1.048e-4	1.090e+6	5.355
phase + valence + drug + genotype + phase × valence + phase × drug + valence × drug + phase × genotype + drug × genotype + phase × drug × genotype	0.006	4.337e-7	7.199e-5	1.587e+6	7.242
phase + valence + drug + genotype + phase × valence + phase × drug + valence × drug + phase × genotype + drug × genotype + phase × valence × drug	0.006	4.008e-7	6.653e-5	1.717e+6	40.653
phase + valence + drug + genotype + phase × valence + phase × drug + phase × genotype + valence × genotype + drug × genotype + phase × drug × genotype	0.006	3.418e-7	5.673e-5	2.013e+6	25.641
phase + valence + drug + genotype + phase × valence + phase × drug + valence × drug + phase × genotype + valence × genotype	0.006	2.693e-7	4.470e-5	2.555e+6	28.041
phase + valence + drug + genotype + phase × valence + phase × drug + phase × genotype + valence × genotype + phase × valence × genotype	0.006	2.238e-7	3.715e-5	3.075e+6	5.455
phase + valence + drug + genotype + phase × valence + phase × drug + valence × drug + phase × genotype + valence × genotype + drug × genotype + valence × drug × genotype	0.006	1.259e-7	2.090e-5	5.465e+6	6.020
phase + valence + drug + genotype + phase × valence + phase × drug + valence × drug + valence × genotype + phase × valence × drug	0.006	1.178e-7	1.955e-5	5.842e+6	4.499
phase + valence + drug + genotype + phase × valence + phase × drug + valence × drug + valence × genotype + drug × genotype + phase × valence × drug + valence × drug × genotype	0.006	9.145e-8	1.518e-5	7.525e+6	7.362
phase + valence + drug + genotype + phase × valence + valence × drug + phase × genotype + valence × genotype + phase × valence × genotype	0.006	7.921e-8	1.315e-5	8.687e+6	5.540
phase + valence + drug + genotype + phase × valence + phase × drug + phase × genotype + valence × genotype + drug × genotype + phase × valence × genotype	0.006	7.725e-8	1.282e-5	8.907e+6	6.297
phase + valence + drug + genotype + phase × valence + phase × drug + valence × drug + phase × genotype + valence × genotype + drug × genotype	0.006	6.691e-8	1.111e-5	1.028e+7	6.305

Models	P(M)	P(M|data)	BF_M	BF_{01}	error %
phase + valence + drug + genotype + phase × valence + valence × drug + phase × genotype + valence × genotype + drug × genotype + phase × valence × genotype + valence × drug × genotype	0.006	5.644e-8	9.368e-6	1.219e+7	7.881
phase + valence + drug + genotype + phase × valence + phase × drug + valence × drug + valence × genotype + drug × genotype + phase × valence × drug	0.006	4.236e-8	7.032e-6	1.624e+7	4.956
phase + valence + drug + genotype + phase × valence + valence × drug + phase × genotype + valence × genotype + drug × genotype + phase × valence × genotype	0.006	3.789e-8	6.290e-6	1.816e+7	16.974
phase + valence + drug + genotype + phase × valence + phase × drug + valence × drug + phase × genotype + drug × genotype + phase × valence × drug + phase × drug × genotype	0.006	3.425e-8	5.686e-6	2.009e+7	8.194
phase + valence + drug + genotype + phase × valence + phase × drug + valence × drug + phase × genotype + valence × genotype + drug × genotype + phase × drug × genotype + valence × drug × genotype	0.006	1.621e-8	2.690e-6	4.246e+7	5.513
phase + valence + drug + genotype + phase × valence + phase × drug + valence × drug + phase × genotype + valence × genotype + phase × valence × drug	0.006	1.327e-8	2.203e-6	5.184e+7	5.299
phase + valence + drug + genotype + phase × valence + phase × drug + valence × drug + phase × genotype + valence × genotype + drug × genotype + phase × valence × drug + valence × drug × genotype	0.006	1.057e-8	1.755e-6	6.510e+7	7.665
phase + valence + drug + genotype + phase × valence + phase × drug + phase × genotype + valence × genotype + drug × genotype + phase × valence × genotype + phase × drug × genotype	0.006	1.010e-8	1.676e-6	6.815e+7	5.526
phase + valence + drug + genotype + phase × valence + phase × drug + valence × drug + phase × genotype + valence × genotype + drug × genotype + phase × drug × genotype	0.006	8.669e-9	1.439e-6	7.937e+7	6.437
phase + valence + drug + genotype + phase × valence + phase × drug + valence × drug + phase × genotype + valence × genotype + phase × valence × genotype	0.006	8.004e-9	1.329e-6	8.597e+7	6.586
phase + valence + drug + genotype + phase × valence + phase × drug + valence × drug + phase × genotype + valence × genotype + drug × genotype + phase × valence × drug	0.006	6.185e-9	1.027e-6	1.113e+8	11.363
phase + valence + drug + genotype + phase × valence + phase × drug + valence × drug + phase × genotype + valence × genotype + drug × genotype + phase × valence × genotype + valence × drug × genotype	0.006	5.361e-9	8.900e-7	1.283e+8	7.958
phase + valence + drug + genotype + phase × valence + phase × drug + valence × drug + phase × genotype + valence × genotype + drug × genotype + phase × valence × genotype	0.006	2.700e-9	4.482e-7	2.549e+8	8.062
phase + valence + drug + genotype + phase × valence + phase × drug + valence × drug + phase × genotype + valence × genotype + drug × genotype + phase × valence × drug + phase × drug × genotype + valence × drug × genotype	0.006	1.463e-9	2.428e-7	4.704e+8	10.084
phase + valence + drug + genotype + phase × valence + phase × drug + valence × drug + phase × genotype + valence × genotype + phase × valence × drug + phase × valence × genotype	0.006	8.335e-10	1.384e-7	8.256e+8	31.873

Models	P(M)	P(M|data)	BF_M	BF_{01}	error %
phase + valence + drug + genotype + phase × valence + phase × drug + valence × drug + phase × genotype + valence × genotype + drug × genotype + phase × valence × genotype + phase × drug × genotype + valence × drug × genotype	0.006	8.232e-10	1.366e-7	8.359e+8	13.577
phase + valence + drug + genotype + phase × valence + phase × drug + valence × drug + phase × genotype + valence × genotype + drug × genotype + phase × valence × drug + phase × drug × genotype	0.006	6.318e-10	1.049e-7	1.089e+9	5.628
phase + valence + drug + genotype + phase × valence + phase × drug + valence × drug + phase × genotype + valence × genotype + drug × genotype + phase × valence × drug + phase × valence × genotype + valence × drug × genotype	0.006	4.026e-10	6.683e-8	1.709e+9	6.881
phase + valence + drug + genotype + phase × valence + phase × drug + valence × drug + phase × genotype + valence × genotype + drug × genotype + phase × valence × genotype + phase × drug × genotype	0.006	3.516e-10	5.837e-8	1.957e+9	6.922
phase + valence + drug + genotype + phase × valence + phase × drug + valence × drug + phase × genotype + valence × genotype + drug × genotype + phase × valence × drug + phase × valence × genotype	0.006	2.248e-10	3.731e-8	3.061e+9	8.198
phase + valence + drug + genotype + phase × valence + phase × drug + valence × drug + phase × genotype + valence × genotype + drug × genotype + phase × valence × drug + phase × valence × genotype + phase × drug × genotype + valence × drug × genotype	0.006	4.982e-11	8.271e-9	1.381e+10	5.990
phase + valence + drug + genotype + phase × valence + phase × drug + valence × drug + phase × genotype + valence × genotype + drug × genotype + phase × valence × drug + phase × valence × genotype + phase × drug × genotype	0.006	2.602e-11	4.320e-9	2.644e+10	6.285
phase + valence + drug + genotype + phase × valence + phase × drug + valence × drug + phase × genotype + valence × genotype + drug × genotype + phase × valence × drug + phase × valence × genotype + phase × drug × genotype + valence × drug × genotype + phase × valence × drug × genotype	0.006	3.496e-12	5.803e-10	1.968e+11	5.822
phase + valence	0.006	3.284e-34	5.451e-32	2.095e+33	6.007
phase + valence + genotype	0.006	5.078e-35	8.429e-33	1.355e+34	3.887
phase + valence + drug	0.006	4.777e-35	7.929e-33	1.441e+34	3.950
phase + valence + drug + genotype	0.006	8.733e-36	1.450e-33	7.880e+34	5.331
phase + valence + genotype + phase × genotype	0.006	5.623e-36	9.334e-34	1.224e+35	4.085
phase + valence + drug + phase × drug	0.006	5.287e-36	8.776e-34	1.302e+35	6.654
phase + valence + drug + genotype + drug × genotype	0.006	2.736e-36	4.541e-34	2.515e+35	4.530
phase + valence + drug + valence × drug	0.006	1.591e-36	2.641e-34	4.324e+35	4.447
phase + valence + genotype + valence × genotype	0.006	1.232e-36	2.045e-34	5.586e+35	6.154
phase + valence + drug + genotype + phase × genotype	0.006	9.391e-37	1.559e-34	7.327e+35	6.241

Models	P(M)	P(M|data)	BF_M	BF_{01}	error %
phase + valence + drug + genotype + phase × drug	0.006	8.402e-37	1.395e-34	8.189e+35	4.620
phase + valence + drug + genotype + phase × genotype + drug × genotype	0.006	3.118e-37	5.176e-35	2.207e+36	5.762
phase + valence + drug + genotype + phase × drug + drug × genotype	0.006	2.800e-37	4.648e-35	2.458e+36	6.695
phase + valence + drug + genotype + valence × drug	0.006	2.787e-37	4.627e-35	2.469e+36	4.734
phase + valence + drug + genotype + valence × genotype	0.006	1.868e-37	3.101e-35	3.684e+36	4.871
phase + valence + drug + phase × drug + valence × drug	0.006	1.621e-37	2.690e-35	4.246e+36	5.019
phase + valence + genotype + phase × genotype + valence × genotype	0.006	1.216e-37	2.018e-35	5.659e+36	11.437
phase + valence + drug + genotype + valence × drug + drug × genotype	0.006	1.003e-37	1.664e-35	6.862e+36	8.381
phase + valence + drug + genotype + phase × drug + phase × genotype	0.006	9.481e-38	1.574e-35	7.258e+36	12.650
phase + valence + drug + genotype + valence × genotype + drug × genotype	0.006	6.119e-38	1.016e-35	1.124e+37	4.603
phase + valence + drug + genotype + valence × drug + phase × genotype	0.006	3.793e-38	6.297e-36	1.814e+37	14.783
phase + valence + drug + genotype + phase × drug + phase × genotype + drug × genotype	0.006	3.345e-38	5.552e-36	2.057e+37	11.165
phase + valence + drug + genotype + phase × drug + valence × drug	0.006	2.812e-38	4.667e-36	2.447e+37	5.894
phase + valence + drug + genotype + phase × genotype + valence × genotype	0.006	2.109e-38	3.501e-36	3.262e+37	5.635
phase + valence + drug + genotype + phase × drug + valence × genotype	0.006	1.795e-38	2.980e-36	3.834e+37	4.250
phase + valence + drug + genotype + phase × drug + valence × drug + drug × genotype	0.006	1.020e-38	1.693e-36	6.747e+37	8.277
phase + valence + drug + genotype + valence × drug + phase × genotype + drug × genotype	0.006	9.520e-39	1.580e-36	7.228e+37	4.488
phase + valence + drug + genotype + valence × drug + valence × genotype	0.006	6.520e-39	1.082e-36	1.055e+38	8.620
phase + valence + drug + genotype + phase × genotype + valence × genotype + drug × genotype	0.006	6.088e-39	1.011e-36	1.130e+38	4.348
phase + valence + drug + genotype + phase × drug + valence × genotype + drug × genotype	0.006	5.969e-39	9.909e-37	1.153e+38	9.137
phase + valence + drug + genotype + phase × drug + phase × genotype + drug × genotype + phase × drug × genotype	0.006	3.932e-39	6.527e-37	1.750e+38	6.647
phase + valence + drug + genotype + phase × drug + valence × drug + phase × genotype	0.006	3.750e-39	6.226e-37	1.835e+38	17.764
phase + valence + drug + genotype + valence × drug + valence × genotype + drug × genotype + valence × drug × genotype	0.006	2.132e-39	3.539e-37	3.228e+38	5.194
phase + valence + drug + genotype + valence × drug + valence × genotype + drug × genotype	0.006	1.992e-39	3.307e-37	3.455e+38	5.083
phase + valence + drug + genotype + phase × drug + phase × genotype + valence × genotype	0.006	1.881e-39	3.122e-37	3.659e+38	4.689
phase + valence + drug + genotype + phase × drug + valence × drug + phase × genotype + drug × genotype	0.006	9.420e-40	1.564e-37	7.305e+38	5.114
phase + valence + drug + genotype + valence × drug + phase × genotype + valence × genotype	0.006	6.741e-40	1.119e-37	1.021e+39	7.507
phase + valence + drug + genotype + phase × drug + phase × genotype + valence × genotype + drug × genotype	0.006	6.534e-40	1.085e-37	1.053e+39	5.106

Models	P(M)	P(M|data)	BF_M	BF_{01}	error %
phase + valence + drug + genotype + phase × drug + valence × drug + valence × genotype	0.006	5.806e-40	9.638e-38	1.185e+39	4.618
phase + valence + drug + genotype + valence × drug + phase × genotype + valence × genotype + drug × genotype	0.006	2.558e-40	4.247e-38	2.690e+39	11.176
phase + valence + drug + genotype + valence × drug + phase × genotype + valence × genotype + drug × genotype + valence × drug × genotype	0.006	2.248e-40	3.732e-38	3.060e+39	5.094
phase + valence + drug + genotype + phase × drug + valence × drug + valence × genotype + drug × genotype	0.006	2.108e-40	3.499e-38	3.265e+39	10.081
phase + valence + drug + genotype + phase × drug + valence × drug + valence × genotype + drug × genotype + valence × drug × genotype	0.006	2.020e-40	3.354e-38	3.406e+39	5.211
phase + valence + drug + genotype + phase × drug + phase × genotype + valence × genotype + drug × genotype + phase × drug × genotype	0.006	1.881e-40	3.123e-38	3.657e+39	27.320
phase + valence + drug + genotype + phase × drug + valence × drug + phase × genotype + drug × genotype + phase × drug × genotype	0.006	1.379e-40	2.289e-38	4.990e+39	7.786
phase + valence + drug + genotype + phase × drug + valence × drug + phase × genotype + valence × genotype	0.006	7.411e-41	1.230e-38	9.285e+39	12.396
phase + valence + drug + genotype + phase × drug + valence × drug + phase × genotype + valence × genotype + drug × genotype + valence × drug × genotype	0.006	2.367e-41	3.929e-39	2.907e+40	8.083
phase + valence + drug + genotype + phase × drug + valence × drug + phase × genotype + valence × genotype + drug × genotype	0.006	2.338e-41	3.882e-39	2.943e+40	12.551
phase + valence + drug + genotype + phase × drug + valence × drug + phase × genotype + valence × genotype + drug × genotype + phase × drug × genotype	0.006	1.128e-41	1.872e-39	6.101e+40	72.375
phase + valence + drug + genotype + phase × drug + valence × drug + phase × genotype + valence × genotype + drug × genotype + phase × drug × genotype + valence × drug × genotype	0.006	2.891e-42	4.799e-40	2.380e+41	6.389
valence	0.006	1.455e-48	2.415e-46	4.730e+47	3.607
valence + genotype	0.006	2.419e-49	4.016e-47	2.844e+48	3.973
valence + drug	0.006	2.332e-49	3.871e-47	2.950e+48	4.105
valence + drug + genotype	0.006	3.899e-50	6.473e-48	1.765e+49	5.200
valence + drug + genotype + drug × genotype	0.006	1.212e-50	2.013e-48	5.676e+49	4.192
valence + drug + valence × drug	0.006	7.131e-51	1.184e-48	9.649e+49	3.977
valence + genotype + valence × genotype	0.006	5.288e-51	8.779e-49	1.301e+50	3.857
valence + drug + genotype + valence × drug	0.006	1.231e-51	2.044e-49	5.588e+50	4.954
valence + drug + genotype + valence × genotype	0.006	8.222e-52	1.365e-49	8.369e+50	5.536
valence + drug + genotype + valence × drug + drug × genotype	0.006	3.727e-52	6.187e-50	1.846e+51	4.126

Models	P(M)	P(M|data)	BF_M	BF_{01}	error %
valence + drug + genotype + valence × genotype + drug × genotype	0.006	2.760e-52	4.581e-50	2.493e+51	5.461
valence + drug + genotype + valence × drug + valence × genotype	0.006	2.574e-53	4.273e-51	2.673e+52	4.309
valence + drug + genotype + valence × drug + valence × genotype + drug × genotype	0.006	8.393e-54	1.393e-51	8.198e+52	8.132
valence + drug + genotype + valence × drug + valence × genotype + drug × genotype + valence × drug × genotype	0.006	6.659e-54	1.105e-51	1.033e+53	4.964
phase	0.006	3.500e-217	5.809e-215	1.966e+216	3.629
phase + genotype	0.006	4.057e-218	6.734e-216	1.696e+217	4.349
phase + drug	0.006	3.800e-218	6.307e-216	1.811e+217	4.232
phase + drug + genotype	0.006	4.428e-219	7.350e-217	1.554e+218	5.172
phase + genotype + phase × genotype	0.006	4.072e-219	6.760e-217	1.690e+218	4.325
phase + drug + phase × drug	0.006	3.782e-219	6.277e-217	1.820e+218	6.810
phase + drug + genotype + drug × genotype	0.006	9.088e-220	1.509e-217	7.572e+218	4.082
phase + drug + genotype + phase × genotype	0.006	4.260e-220	7.071e-218	1.615e+219	5.524
phase + drug + genotype + phase × drug	0.006	3.865e-220	6.416e-218	1.780e+219	4.052
phase + drug + genotype + phase × genotype + drug × genotype	0.006	9.417e-221	1.563e-218	7.307e+219	4.993
phase + drug + genotype + phase × drug + drug × genotype	0.006	8.624e-221	1.432e-218	7.979e+219	4.225
phase + drug + genotype + phase × drug + phase × genotype	0.006	4.079e-221	6.771e-219	1.687e+220	6.297
phase + drug + genotype + phase × drug + phase × genotype + drug × genotype	0.006	9.504e-222	1.578e-219	7.240e+220	7.630
Null model (incl. subject)	0.006	1.779e-222	2.954e-220	3.867e+221	3.561
phase + drug + genotype + phase × drug + phase × genotype + drug × genotype + phase × drug × genotype	0.006	1.536e-222	2.550e-220	4.480e+221	29.329
genotype	0.006	1.980e-223	3.287e-221	3.475e+222	3.707
drug	0.006	1.858e-223	3.085e-221	3.703e+222	4.339
drug + genotype	0.006	2.058e-224	3.417e-222	3.343e+223	4.009
drug + genotype + drug × genotype	0.006	4.628e-225	7.683e-223	1.487e+224	4.320

Legend: P(M): prior model probability, P(M|data): posterior model probability, BF_M: posterior model odds, BF_{01}: evidence for the best model (first row) relative to alternative model, error %: error of the Gaussian quadrature integration routine used for the computation of the Bayes factor.

Supplementary Table 18: Analysis of effects of Bayesian ANOVA for ACP key press speed

Effects	P(incl)	P(excl)	P(incl|data)	P(excl|data)	BF_{excl}
phase	0.886	0.114	1.000	1.299e-14	1.012e-13
valence	0.886	0.114	1.000	1.299e-14	1.012e-13
drug	0.886	0.114	0.168	0.832	38.514
genotype	0.886	0.114	0.181	0.819	35.266
phase × valence	0.503	0.497	1.000	1.299e-14	1.315e-14
phase × drug	0.503	0.497	0.016	0.984	62.705
phase × genotype	0.503	0.497	0.019	0.981	52.729
valence × drug	0.503	0.497	0.006	0.994	160.330
valence × genotype	0.503	0.497	0.005	0.995	206.605
drug × genotype	0.503	0.497	0.010	0.990	98.025
phase × valence × drug	0.120	0.880	4.053e-5	1.000	3356.633
phase × valence × genotype	0.120	0.880	2.326e-5	1.000	5850.071
phase × drug × genotype	0.120	0.880	1.197e-5	1.000	11362.580
valence × drug × genotype	0.120	0.880	1.588e-5	1.000	8567.455
phase × valence × drug × genotype	0.006	0.994	3.496e-12	1.000	1.723e+9

Legend: P(incl): prior inclusion probability, P(excl): prior exclusion probability, P(incl|data): posterior inclusion probability, P(excl|data): posterior exclusion probability, BF_{excl}: exclusion Bayes factor.

Supplementary Table 19: Descriptive statistics of experimental effects for the nicotine and genotype groups

	Nicotine		Placebo	
	9R	10/10	9R	10/10
SBR (N/s)				
	0.17 (0.17)	0.18 (0.18)	0.16 (0.14)	0.17 (0.19)
SPEM gain				
0.2 Hz, no background	0.96 (0.06)	0.96 (0.09)	0.94 (0.07)	0.93 (0.09)
0.2 Hz, background	0.87 (0.15)	0.86 (0.17)	0.86 (0.14)	0.85 (0.14)
0.4 Hz, no background	0.88 (0.12)	0.88 (0.15)	0.87 (0.1)	0.86 (0.12)
0.4 Hz, background	0.77 (0.21)	0.75 (0.23)	0.75 (0.2)	0.71 (0.19)
0.6 Hz, no background	0.78 (0.17)	0.79 (0.19)	0.79 (0.15)	0.77 (0.16)
0.6 Hz, background	0.6 (0.25)	0.61 (0.26)	0.62 (0.24)	0.57 (0.23)
Stop signal SSRT				
	210.82 (42.26)	221.19 (42.61)	214.52 (32.73)	216.13 (30.77)
Go RT				
simple choice task	368.12 (34.93)	370.45 (31.46)	382.55 (33.63)	375.94 (32.44)
stop signal task	521.21 (121.09)	487.71 (118.74)	540.04 (132.02)	529.34 (124.00)
ACP button presses (N/s)				
anticipatory phase, negative	5.06 (2.13)	5.29 (2.13)	5.05 (2.75)	4.83 (2.05)
anticipatory phase, neutral	1.22 (1.21)	0.95 (0.88)	1.16 (1.74)	1.15 (1.36)
anticipatory phase, positive	3.76 (1.93)	4.15 (1.87)	4.14 (2.63)	3.69 (2.34)
consummatory phase, negative	4.18 (1.83)	4.46 (1.64)	4.46 (1.83)	3.87 (1.58)
consummatory phase, neutral	0.83 (0.93)	0.65 (0.59)	0.85 (0.97)	0.75 (0.82)
consummatory phase, positive	2.94 (1.71)	3.07 (1.61)	3.27 (1.78)	2.87 (1.78)

Legend: Numbers indicate mean and standard deviations (in brackets). SBR = spontaneous blink rate, SSRT = stop signal reaction time, RT = reaction time, ACP = Anticipatory and Consummatory Pleasure task.
